# Heterologous Hydrogenase Overproduction Systems for Biotechnology—An Overview

**DOI:** 10.3390/ijms21165890

**Published:** 2020-08-16

**Authors:** Qin Fan, Peter Neubauer, Oliver Lenz, Matthias Gimpel

**Affiliations:** 1 Institute of Biotechnology, Technical University of Berlin, Ackerstraße 76, 13355 Berlin, Germany; qin.fan@campus.tu-berlin.de (Q.F.); peter.neubauer@tu-berlin.de (P.N.); 2 Department of Chemistry, Technical University of Berlin, Straße des 17. Juni 135, 10623 Berlin, Germany; oliver.lenz@tu-berlin.de

**Keywords:** hydrogenases, heterologous expression systems, metalloprotein, biohydrogen, renewable energy, oxygen-tolerance, difficult-to-express protein, in vitro maturation

## Abstract

Hydrogenases are complex metalloenzymes, showing tremendous potential as H_2_-converting redox catalysts for application in light-driven H_2_ production, enzymatic fuel cells and H_2_-driven cofactor regeneration. They catalyze the reversible oxidation of hydrogen into protons and electrons. The apo-enzymes are not active unless they are modified by a complicated post-translational maturation process that is responsible for the assembly and incorporation of the complex metal center. The catalytic center is usually easily inactivated by oxidation, and the separation and purification of the active protein is challenging. The understanding of the catalytic mechanisms progresses slowly, since the purification of the enzymes from their native hosts is often difficult, and in some case impossible. Over the past decades, only a limited number of studies report the homologous or heterologous production of high yields of hydrogenase. In this review, we emphasize recent discoveries that have greatly improved our understanding of microbial hydrogenases. We compare various heterologous hydrogenase production systems as well as in vitro hydrogenase maturation systems and discuss their perspectives for enhanced biohydrogen production. Additionally, activities of hydrogenases isolated from either recombinant organisms or in vivo/in vitro maturation approaches were systematically compared, and future perspectives for this research area are discussed.

## 1. Introduction

The world’s still increasing hunger for energy and the resulting excessive use of fossil fuels is a major reason for environmental pollution and global warming. These problems are one of the most urgent and actual questions of humankind. Therefore, the search for and utilization of so-called alternative energy sources is an important task. A promising solution is the utilization of “green” molecular hydrogen (H_2_) as it contains a high energy potential and its combustion releases only water [1,2,3,4]. Unfortunately, the already well-established non-biological systems for the production, storage and utilization of H_2_ rely on noble metal catalysts, which are resource-limited, expensive, and can easily be destroyed by, e.g., CO or H_2_S impurities in commercially available H_2_ gas [5,6,7]. H_2_-converting biocatalysts, the so-called hydrogenases, however, utilize abundant metals, such as iron and nickel, for catalysis and are often resistant towards CO or H_2_S.

During evolution, the harsh environments and low substrate availability lead to the development of highly specific, but robust hydrogenases with sometimes extremely high affinity towards hydrogen [8]. Based on the metal ion composition of their active center, hydrogenases can be divided into the three classes of [Fe]-, [FeFe]- and [NiFe]-hydrogenases [9]. In contrast to the FeS cluster-free [Fe]-hydrogenases, most [FeFe]- and [NiFe]-hydrogenases are more complex and display a multisubunit architecture where the active site is deeply buried within the catalytic subunit. In many cases, electron transport is mediated by almost linearly arranged FeS clusters located in one or more further subunits [9]. In all cases, the catalytic subunits are synthesized in an apo-form that subsequently receives the metallocofactor from a dedicated maturation machinery to form the enzymatically active holo-enzyme. In case of [FeFe]-hydrogenases, this maturation machinery encompasses three different maturases, while [Fe]- and [NiFe]-hydrogenases require at least seven and six, respectively, auxiliary proteins involved in active site assembly and incorporation [10,11].

Biological H_2_ production is a promising alternative to industrial H_2_ synthesis from fossil fuel. In recent decades, hydrogenases have been widely explored for their potential application in biotechnology [12,13]. Hydrogenases can be applied for the construction of hydrogenase-photosystem hybrid complexes for the evolution of H_2_ [14,15]. Apart from that, the H_2_ oxidation activity of hydrogenases has been exploited in electrochemical fuel cells, which are independent of the traditional platinum catalysts [16]. Furthermore, hydrogenases have been successfully used in H_2_-driven co-factor regeneration systems to recycle co-factors such as NAD(H) and NADP(H), which are required for many oxidoreductases of commercial interest [17,18,19]. These biotechnological applications of hydrogenases are, however, still in their infancy [20,21]. Additionally, the relatively low hydrogenase content in the native hosts impedes their purification in sufficient amounts for a detailed analysis of their structure/function and for biotechnological applications. The heterologous production of hydrogenase in well-studied expression hosts such as *Escherichia coli* might be a solution. Even though the *E. coli* genome has the coding capacity of four different indigenous hydrogenases [22], owing to the complexity of the post-translational maturation and the high susceptibility of the metal centers to inhibitory O_2_, heterologous production of active hydrogenase with high purity and in large quantities remains challenging. Nevertheless, ever since the hydrogenase maturation pathways had been deciphered [11,23,24], numerous approaches for the recombinant production of hydrogenases were realized. The availability of a number of different hydrogenases enabled a deep understanding of their catalytic mechanisms, and the groundwork was laid for their biotechnological application [9,25].

This review summarizes recent advances in the development of heterologous hydrogenase production systems. We also discuss in vitro reconstitution systems for the production of functional active hydrogenases from apo-enzymes and chemically synthesized cofactors.

## 2. [Fe]-Hydrogenase Production Systems

[Fe]-hydrogenases can only be found in some methanogenic archaea. Apart from the other two classes, they do not contain iron-sulfur clusters. The most widely studied [Fe]-hydrogenase is Hmd from *Methanothermobacter marburgensis*. Hmd catalyzes the reversible, H_2_-driven reduction of methenyl-tetrahydromethanopterin (methenyl-H_4_MPT^+^) with H_2_ to methylene-H_4_MPT and H^+^, an intermediate step during the reduction of CO_2_ to methane [26]. Hmd is a homodimer [27] and harbors a unique iron-guanylyl pyridinol (FeGP) cofactor at the active site that is typical for [Fe]-hydrogenases. The FeGP cofactor contains a cysteine thiol, two CO, one acyl-carbon and one hybridized pyridinol nitrogen [3,28,29,30,31].

Active Hmd proteins were first purified from their native hosts, including *M. marburgensis* (formerly *Methanobacterium thermoautotrophicum* [27]), *Methanocaldococcus jannaschii* [31] and *Methanopyrus kandleri* [32] with specific activities of 1500, 350 and 360 U mg^−1^, respectively (Table 1). The hydrogenases were purified under strictly anoxic conditions in the dark, and the enzymes proved to be stable for several hours in air. Their activity was inactivated by dioxygen only in the presence of reducing substrates, such as methylene-H_4_MPT or methenyl-H_4_MPT/H_2_ via a decomposition of the FeGP cofactor by H_2_O_2_ generated from O_2_ [33]. However, Hmd and its FeGP cofactor were inactivated upon exposure to UV-A (320–400 nm) or blue-light (400–500 nm), which is a unique feature among the three types of hydrogenases [34]. This sensitivity to light and their ability to transfer only hydride ions but not electrons are the main obstacles to the use of these enzymes in technological applications [26].

**Figure 1:**
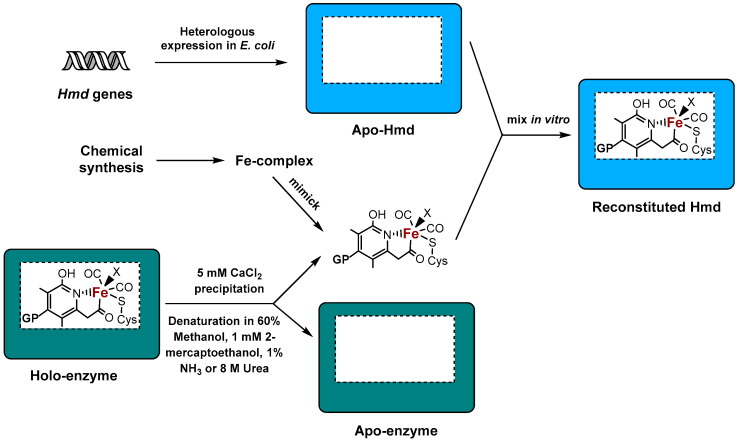
Artificial reconstitution of the active FeGP cofactor of [Fe]-hydrogenase in vitro. Active iron-guanylyl pyridinol (FeGP) cofactor is extracted in an intact form from the active site of a native [Fe]-hydrogenase by denaturation under the indicated conditions. The unfolded protein is removed by salt precipitation to obtain highly purified FeGP cofactor. The active reconstituted holoenzyme with a fully assembled active site is formed by mixing of the isolated apoenzyme heterologously produced in *E. coli* and the extracted FeGP cofactor. In addition to this, many Fe-complexes have been chemically synthesized to mimic the active site of [Fe]-hydrogenase. These complexes become active only when incorporated into the apo-enzyme of Fe-hydrogenases forming so-called semi-synthetic [Fe]-hydrogenases. X represents a solvent molecule that is bound at the “open” site of the crystal structure of the native enzyme. GP: guanylylpyridinol.

Recent studies showed that the reconstitution of active [Fe]-hydrogenase in vitro is feasible (Figure 1). However, since chemical synthesis of the FeGP cofactor was not possible so far, the cofactor had to be extracted from native [Fe]-hydrogenases. The inactive [Fe]-hydrogenase apo-enzymes from some methanogens have been successfully produced in *E. coli* either as soluble proteins or in the form of inclusion bodies [36]. To obtain an active cofactor, Shima et al. [36] optimized the extraction process, yielding purified FeGP cofactor from *M. marburgensis*, which had the ability to restore a specific activity of approx. 700 U from 1 mg of enzyme. This activity is actually higher than that of purified [Fe]-hydrogenase (400–600 U mg^−1^) [34]. The activity of the reconstituted Hmd from *M. jannaschii* (300–400 U mg^−1^) is equivalent to the full activity of the Hmd purified from *M. marburgensis* (400–600 U mg^−1^) [34]. Remarkably, the inactive apo-Hmd from *M. jannaschii* was reactivated up to a specific activity of 1100 U mg^−1^ by the addition of the FeGP cofactor purified via ultrafiltration from active Hmd from *M. marburgensis*, which had been denatured in 8 M urea instead of MeOH, 2-mercaptoethanol and ammonia solution to release the cofactor [31]. However, the difference in *Mja*Hmd activity during activation with purified FeGP is mainly attributed to the different reaction temperatures used for the activity measurements, while 1100 U mg^−1^ were obtained at 65 °C [31], the measurement at 40 °C resulted in 300–400 U mg^−1^ Hmd activity [34]. Similarly, heterologously produced Hmd from *M. kandleri* was activated by the purified FeGP cofactors from *M. jannaschii* or *M. kandleri* [31]. These studies clearly showed that the cofactor is released in an intact form upon enzyme denaturation in the presence of urea and thiol reagents. At least seven *hmd* co-occurring genes (*hcgA-G*) are required for FeGP cofactor synthesis [30,38,39]. So far, the functions of five of them, HcgB, HcgC, HcgD, HcgE and HcgF, in FeGP cofactor biosynthesis have been elucidated [40,41,42,43,44]. The functions of HcgA and HcgG are, however, still not known, and only a putative role in CO ligand synthesis or pyridinol assembly is proposed [42]. The seven Hcg proteins are, in contrast to the Hmd-paralogue HmdII, essential for Fe hydrogenase maturation [39]. Nevertheless, Goldman et al. suggested a function of HmdII as a scaffold for the synthesis of the FeGP cofactor [45], while Fujishiro et al. excluded a scaffold function and proposed that HmdII could act as a sensor of intracellular methylene H4MPT concentration and thus be involved in the regulation of *hmd* gene expression [46]. However, further experimental evidence is required in both cases.

Native hydrogenases exclusively utilize Fe and/or Ni for H_2_ activation. However, other transition metals are also known to activate or catalyze the production of hydrogen in synthetic chemical systems. Consequently, a non-native metal hydrogenase has been constructed by incorporation of a Mn-complex into the apo-[Fe]-hydrogenase from *M. jannaschii* heterologously produced in *E. coli*, resulting in a [Mn]-hydrogenase with a comparable activity to semi-synthetic [Fe]-hydrogenase [37,47]. However, compared to the hydrogenase reconstituted with the native FeGP cofactor the semi-synthetic enzymes showed significant less activity. This might be attributed to the lack of the guanosine monophosphate (GMP) moiety and two methyl groups in the pyridinol group of the synthetic cofactors used for reconstitution of both semi-synthetic [Fe]- and [Mn]-hydrogenase. Nevertheless, the apo-enzyme reconstituted with the Mn(I)-complex exhibits the highest activity and broadest scope in catalytic hydrogenation among all engineered variants [37]. Compared to other synthetic Mn catalysts, the semisynthetic [Mn]-hydrogenase displays a unique activity in Mn-catalyzed hydrogenation of compounds analogous to the natural substrate of [Fe]-hydrogenase, methenyl-H_4_MPT^+^ [37]. These results demonstrate the potential use of biomimetic catalysts in hydrogenation reactions.

Since their discovery about 30 years ago, [Fe]-hydrogenases are still the least studied hydrogenases, but they have uncovered new structural and mechanistic surprises that often required the re-assessment of the proposed catalytic mechanism [48,49,50]. Apart from that, research on [Fe]-hydrogenases is still conducted in only a few research groups, which is in marked contrast to the situation for [FeFe]- and [NiFe]-hydrogenases.

## 3. [FeFe]-Hydrogenase Production Systems

**Figure 2:**
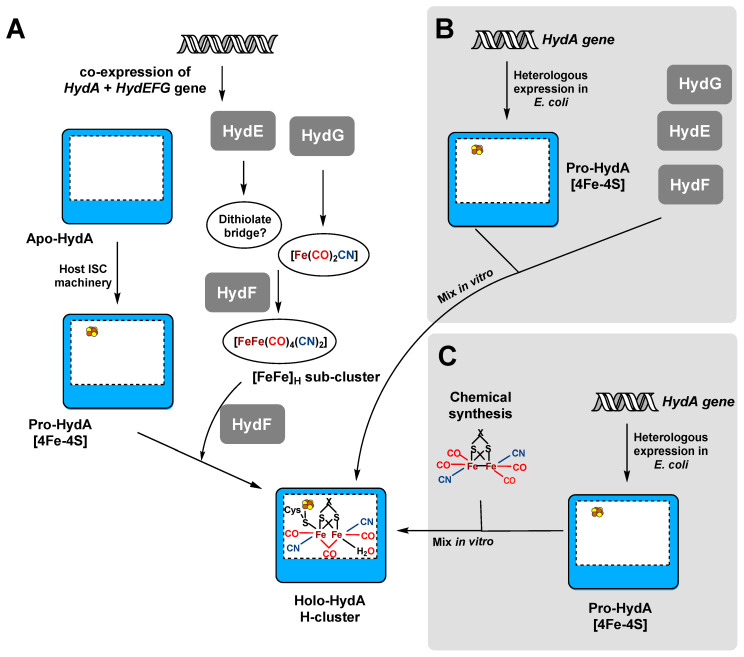
Heterologous production of active [FeFe]-hydrogenases in vivo and in vitro. (**A**) In vivo production of the active [FeFe]-hydrogenases requires the co-expression of specific maturases. First, the iron-sulfur cluster is inserted into the apo-enzyme by the host’s universal ISC (iron-sulphur cluster assembly machinery) pathway producing an inactive pro-enzyme. In the second step, the [FeFe]_H_ sub-cluster precursor is produced by the co-synthesized maturases HydE and HydG, while the third maturase, HydF, serves as scaffold protein, which also inserts the cofactor into Pro-HydA. This results in active [FeFe] hydrogenase. The question mark denotes unknown function(s). **(B)** Artificial in vitro maturation is achieved by the heterologous production of the apo-hydrogenase and the three maturases and subsequent mixing of the purified proteins. (**C**) Alternatively, artificial in vitro maturation can be achieved by the heterologous production of the apo-hydrogenase and subsequent mixing of the purified protein with a chemically synthesized [2Fe] subcluster. The bridging ligand X might be NH/NH_2_^+^ in the native maturation or additionally CH_2_, or O when using artificial maturation.

[FeFe]-hydrogenases are widespread in strictly anaerobic bacteria [51], fungi, protists [52,53] as well as in some unicellular green algae [54,55]. Consequently, they are synthesized under strictly anoxic conditions [9]. [FeFe]-hydrogenases occur as monomeric species, but hetero-dimeric, -trimeric and -tetrameric protein are also known [56,57,58,59]. They harbor a unique catalytic site, the so-called H-cluster. Many of them also contain additional iron-sulfur clusters [56]. The H-cluster comprises a di-iron [FeFe] sub-cluster, which is equipped with carbonyl and cyanide ligands and connected to a [4Fe-4S] cluster via a bridging cysteine residue (Figure 2A) [56,60].

As early as in the 1980s, Voordouw and coworkers tried to heterologously express the periplasmic [FeFe]-hydrogenase from *Desulfovibrio vulgaris* in *E. coli*, resulting in an inactive hydrogenase lacking the catalytic H-cluster with a yield of about 5% *w/w* of total protein [61,62]. However, to date, the successful (over)production of several [FeFe]-hydrogenases has been achieved by using homologous as well as heterologous hosts (summarized in Table 2).

### 3.1. Recombinant [FeFe] Hydrogenase Production in the Presence of the Maturases HydE, F and G

Initial attempts to heterologously produce functional [FeFe]-hydrogenases had only limited success, due to the limited understanding of their maturation. The heterologous hosts were not capable of synthesizing the complete H-cluster, resulting in either catalytically inactive apo-enzymes [61,90] or in protein with only very low activity [74,91]. In 2004, Posewitz and coworkers produced *Chlamydomonas reinhardtii* HydA1 in *E. coli* and co-expressed the *hydEFG* genes encoding the maturases from *C. reinhardtii* [78]. The purified recombinant *Cre*HydA1 displayed catalytic activity, which was, however, ca. 3-fold lower than the activity of the enzyme isolated from its native host. Despite the low expression levels of the *C. reinhardtii* genes, this study demonstrated impressively that the three maturases, HydF, HydE and HydG, are essential for the maturation and integration of the H-cluster to produce active [FeFe]-hydrogenase (Figure 2A). Later, the production of *Cre*HydA1 was improved by using *Clostridium acetobutylicum* as a heterologous expression host. With this strategy, functional *Cre*HydA with a final activity of about 760 µmol H_2_/(min·mg) was obtained [76]. Moreover, King et al. co-expressed the *C. acetobutylicum* maturase genes *hydEFG* with the genes encoding the monomeric [FeFe]-hydrogenases from *C. acetobutylicum*, *C. reinhardtii*, *Clostridium pasteurianum* and *Clostridium saccharobutylicum* in *E. coli*, yielding stable and active HydA enzymes with 10-fold higher productivity compared to the native hosts [64,92]. The purification process has also been improved over the years. [FeFe]-hydrogenase activity can markedly decrease during storage of the cells, storage of the purified proteins and during purification. Hence, Girbal et al. [76] introduced modifications into the hydrogenase purification protocol. In this, optimized protocol cells are flushed with pure H_2_ at the end of the cultivation to prevent oxidative damage of the protein. Furthermore, cell disruption was directly performed after the cultivation with freshly harvested cells. With this optimized purification protocol, Demuez et al. [63] increased the specific activities of *C. acetobutylicum* [FeFe]-hydrogenase by 16-fold for H_2_ production and by 130-fold for H_2_ oxidation.

Based on the beneficial codon usage and its hydrogenase maturation ability, Sybirna et al. [82] found that *Shewanella oneidensis* is a suitable host for heterologous production of functional [FeFe]-hydrogenase from *C. reinhardtii*. *S. oneidensis* is a facultatively anaerobic, Gram-negative γ-proteobacterium that possesses a [FeFe]-hydrogenase operon [93]. Compared to the production of *Cre*HydA1 in *C. acetobutylicum* [76], heterologous production in *S. oneidensis* resulted in a similar specific hydrogenase activity but a 5-fold higher enzyme yield [82]. By co-expression of *S. oneidensis hydEFG* during *Cre*HydA1 production in *E. coli*, an even 75-fold higher enzyme yield was obtained without any loss of enzyme activity [70]. Co-expression of *S. oneidensis hydEFG* maturation proteins was also successfully demonstrated for heterologous production of functional [FeFe]-hydrogenases from *C. pasteurianum* (Hyd1) and *Caldanaerobacter subterranus* (HydABCD) in *E. coli* [70,85]. Additionally, expression of the *S. oneidensis* [FeFe]-hydrogenase operon, including the structural and maturation genes, in *Anabaena* sp. *PCC7120* resulted in heterologous production of active *Son*HydA [89]. In contrast, *Son*HydA was only produced in an inactive form in both *E. coli* and *Anabaena* sp. *PCC 7120* without the co-expression of its own maturation proteins [87], thus further demonstrating the importance of the co-production of specific maturation proteins for successful heterologous production of functional [FeFe]-hydrogenases.

### 3.2. In Vitro Maturation Systems for [FeFe]-Hydrogenases

An alternative to the in vivo production of active [FeFe]-hydrogenases is the in vitro maturation and activation of in vivo produced inactive apo-enzymes using independently produced maturases (Figure 2B) or chemically synthesized di-iron cluster (Figure 2C) [69,73,81,84,94]. An in vitro, cell-free maturation has been developed to generate fully active [FeFe]-hydrogenase. To this end, both *C. saccharobutylicum* pro-HydA and the maturases HydEFG from *C. acetobutylicum* were individually produced in *E. coli*, and subsequently all *E. coli* cell extracts were mixed in a reaction tube, yielding active *Csa*HydA [84]. Furthermore, HydEFG from *S. oneidensis* were used in vitro to activate hydrogenases HydA and HydI from *C pasteurianum* but with varying success. Whereas *Cpa*HydA was produced with only 20% of its original activity, *Cpa*HydI was obtained with a 2-fold higher specific activity compared to the production in *E. coli* in the presence of the *S. oneidensis* maturation proteins [69,72]. In contrast, apo-HydA from *C. reinhardtii* was in vitro activated by the addition of a chemically synthesized di-iron cluster either bound to *Thermotoga maritima* HydF [81] or even in the absence of any auxiliary protein [73]. In both cases, the addition of the chemically synthesized co-factor was sufficient to restore hydrogenase activity comparable to the native hydrogenases [73,81].

*E. coli* can be used for the recombinant production of [FeFe]-hydrogenase apoenzymes from essentially any organism in high yield and purity. The subsequent use of in vitro maturation systems allows to produce active enzymes on demand by mixing the apo-enzyme with any desired set of maturation enzymes or chemically synthesized cofactors. This approach can also be applied for rapid randomized mutant library screening with regard to desired properties, e.g., improved catalytic activity or decreased O_2_-sensitivity [71,80]. Taken together, these studies demonstrate that the in vitro production of active [FeFe]-hydrogenases is possible even in the absence of the otherwise essential specific maturases.

### 3.3. [FeFe]-Hydrogenase Production in Cyanobacteria and Microalgae

In most cases, [FeFe]-hydrogenases are irreversibly inactivated by trace amounts of O_2_ [95,96,97]. This O_2_-sensitivity is one of the most critical drawbacks that hamper the biotechnological use of recombinant hydrogenases. Consequently, several recent studies focus on exploiting particularly more O_2_-tolerant enzymes to improve the understanding of the hydrogenase biogenesis and their catalytic mechanisms in vivo and in vitro.

Recently, cyanobacteria were successfully applied for the heterologous production of [FeFe]-hydrogenases with improved O_2_-tolerance [98]. Asada et al. expressed the hydrogenase I gene from *C. pasteurianum* under control of a strong promoter in the cyanobacterium *Synechococcus* sp. *PCC7924*, which naturally encodes a [NiFe]-hydrogenase [74]. This resulted in a significant increase in hydrogen production compared to wild-type *Synechococcus strain* even without co-expression of maturation genes [74]. Nevertheless, compared to the heterologous production of the enzyme in *E. coli* with co-expression of the maturase genes, the specific activity of the clostridial hydrogenase produced in *Synechococcus* is very low [45,48]. Moreover, a *C. acetobutylicum* [FeFe]-hydrogenase was produced in the cyanobacterium *S. elongatus* sp. *7942* upon co-expression of the *C. reinhardtii hydEFG* genes and *C. acetobutylicum* ferredoxin with, however, again very low specific activity [66]. Berto et al. [83] produced the active [FeFe]-hydrogenase from *C. reinhardtii* in the cyanobacterium *Synechocystis* sp. *PCC6803* without the co-expression of maturase genes and also without significant activity. Taken together, these results show that cyanobacteria are able to synthesize and correctly maturate a functional [FeFe]-hydrogenase in the presence [66] and maybe even in the absence of exogenous auxiliary maturases [83]. Their ability to enable the correct maturation of [FeFe] hydrogenases in the absence of co-expressed auxiliary maturase genes has to be considered as an interesting exception in heterologous hydrogenase production and an alternative for industrial applications.

The photobiological H_2_ production by unicellular green algae has recently become particularly attractive as the raw materials required for this process, solar energy and water, are available in sufficient quantities and at low cost [99,100,101]. Even though, the expression of recombinant hydrogenases in green algae is a promising alternative to bacterial expression, this process is, however, exposed to greater challenges due to O_2_-presence and difficulties in genetic manipulation [102,103]. Recent studies have focused on the use of photosynthetic model organisms, such as *C. reinhardtii* for the production of [FeFe]-hydrogenases thereby coupling the photosynthetic transport chain via ferredoxin with H_2_ production. Among all hydrogenases, the algal HydA shows an excellent H_2_ production activity of up to 8 mmol H_2_/(min·mg) [99]. Additionally, some progress in the recombinant production of natural and engineered [FeFe]-hydrogenases has been achieved through optimizing methods of nuclear and chloroplast transformation of *C. reinhardtii*, e.g., by bead milling, particle bombardment or physical disruption of the cell wall [104,105,106,107].

As discussed above, to date the maturation process allowing the H-cluster assembly is still incompletely characterized. Nevertheless, although further work is needed, the controllable expression of hydrogenase genes in green algae and the coordinated development of light-driven hydrogen production and post-translational processing of hydrogenases is interesting for potential biotechnological and economical applications.

## 4. [NiFe]-Hydrogenase Production Systems

**Figure 3:**
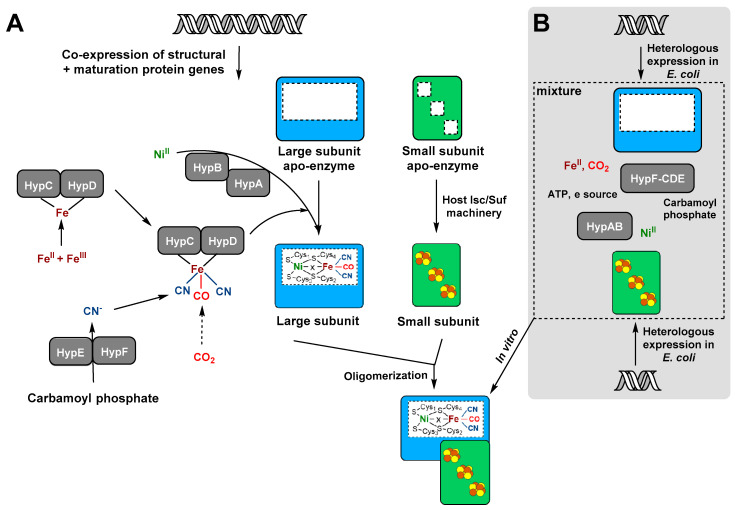
In vivo biosynthesis and in vitro assembly *of* [NiFe]-hydrogenases. (**A**) In vivo maturation of [NiFe]-hydrogenase is achieved by co-expression of specific auxiliary proteins. The FeS clusters of the small subunit are assembled by the universal host Isc/Suf machinery, whereas the catalytic center is synthesized and incorporated into the apo-large subunit with the aid of at least six specific maturases, designated HypA, B, C, D, E, and F. (**B**) In vitro reconstitution of active [NiFe]-hydrogenase by mixing the subunits extracted from the heterologous host *E. coli* and purified maturases, which are essential for the biosynthesis and the insertion of the [NiFe(CN)_2_CO]-active center.

[NiFe]-hydrogenases are widely distributed in all prokaryotes, including archaea and bacteria [108,109]. [NiFe]-hydrogenases are comprised of at least one large subunit (about 60 kDa) containing the [NiFe] active center and one small subunit (about 30 kDa) harboring three almost linearly arranged FeS clusters. The [NiFe] center is deeply buried in the large subunit and linked to the enzyme through four cysteine-derived thiolates. The nickel is coordinated by all four cysteines. Two of them serve as bridging ligands and also coordinated the Fe atom, which is further ligated by a CO and two CN^−^ ligands [110,111]. The two metal ions as well as the diatomic ligands are inserted post-translationally into the apo-hydrogenase through a complex maturation process (Figure 3A) [112]. At least six auxiliary proteins, HypABCDEF, are necessary for biosynthesis of the [NiFe(CN)_2_(CO)] cofactor and its integration into the apo form of the large subunit [11,112]. The maturation systems can be highly specific for their corresponding hydrogenases [9,10]. Thus, efficient heterologous production of functional [NiFe]-hydrogenases is quite challenging.

Nevertheless, several heterologous production systems for functional [NiFe]-hydrogenases have been reported (Table 3), which are discussed in the following section.

### 4.1. Heterologous Production of Hydrogenases in Hosts Encoding Closely Related Native Enzymes

Numerous examples of non-functional recombinant expression of hydrogenase genes have been reported. As early as 1987, the heterodimseric [NiFe]-hydrogenases from *Desulfovibrio vulgaris* Hildenborough has been produced heterologously in *E. coli*, resulting in inactive protein lacking the active site in the large subunit [61]. Later, heterologous expression of [NiFe]-hydrogenase genes from *Acetomicrobium flavidum* [117], *Rhodococcus opacus* [134] and *Synechocystis* sp. PCC6803 [135] was attempted, which also resulted in the production of non-functional hydrogenases.

To this end, recombinant hydrogenase gene expression either in the native hosts or closely related species was considered resulting in the (over-)production of functional hydrogenases. For example, the [NiFe]-hydrogenase from *Desulfovibrio gigas* was successfully produced in an active form in *Desulfovibrio fructosovorans* MR400. The hydrogenase subunits of both species show 64% identity and 80% similarity. However, the specific H_2_ uptake activity of the recombinant *D. gigas* hydrogenase was only 16% of that observed in the native host [119,136]. Transfer of the entire *Rhizobium leguminosarum hup/hyp* gene cluster (*hupSLCDEFGHIJK and hypABFCDEX*) into hydrogenase mutant strains of the closely related α-proteobacteria *Mesorhizobium loti*, *Rhizobium etli* and *Bradyrhizobium japonicum* led to functional HupSL protein with, however, low H_2_ uptake activity [137].

The soluble NAD^+^-reducing [NiFe]-hydrogenase (SH) from the actinomycete *Rhodococcus opacus* was functionally overproduced in the β-proteobacterium *Ralstonia eutropha*, which also hosts an NAD^+^-reducing hydrogenase. Despite the large phylogenetic difference of the two species, their SH proteins are closely related with an overall amino acid identity of these four subunits (HoxFUYH), ranging between 71% and 86%. The recombinantly produced *R. opacus* SH displayed almost 30% of the activity observed for the *R. eutropha* SH [132]. A similar approach was used for successful production of the cytoplasmic NADP^+^-dependent soluble [NiFe]-hydrogenase I (SHI) from *Pyrococcus furiosus* in *E. coli*. Here, thirteen *P. furiosus* genes (four structural and nine maturation genes) were co-expressed in the heterologous host, resulting in a recombinant hydrogenase with half of the specific activity of native *Pfu*SHI [125].

The two peripheral subunits of the membrane-bound [NiFe]-hydrogenase from *Hydrogenovibrio marinus* (*Hma*MBH) show 73% (large subunit) and 78% (small subunit) identity to the corresponding subunits of [NiFe]-hydrogenase 1 (Hyd-1) from *E. coli*. Extracts of *E. coli* cells producing *Hma*MBH showed a H_2_ production activity [121] that was almost 2-fold higher than that of recombinantly overproduced *E. coli* Hyd-1 [120]. Moreover, aerobically purified *Hma*MBH exhibited a 2.4-fold greater specific H_2_ evolution activity than that of recombinant *E. coli* Hyd-1 [120,121].

After the [NiFe]-hydrogenase HynSL from the marine bacterium, *Alteromonas macleodii* (*Ama*HynSL, previously named HyaAB [116]) was successfully produced in aerobically grown, recombinant *A. macleodii* cells [113], Weyman et al. [114] surveyed the genetic elements required for the synthesis of active *Ama*HynSL and HynSL from *Thiocapsa roseopersicina* in aerobically grown *E. coli*. They showed that the accessory *hyp* genes from *A. macleodii* are sufficient to mature HynSL from *T. roseopersicina*, indicating that the assembly machines of the two species must be very similar. Later double-substitution of two residues in the small subunit of *Ama*HynS afforded a 4-fold improvement of its specific H_2_ evolution activity without affecting its hydrogen uptake activity [115]. Following this, comprehensive studies confirmed that the modification of the proximal FeS cluster in HupS improved the H_2_ production by reversing the electron flow within the H_2_-oxidizing hydrogenase HupSL from nitrogen-fixing cyanobacterium *Nostoc punctiforme* [124,138].

The examples described above suggest that a high degree of similarity improves the likelihood of successful production of [NiFe]-hydrogenases in the homologous or closely related heterologous hosts. In most of these cases, co-production of the structural hydrogenase proteins along with their specific maturases is required to achieve correct metal center assembly in the recombinant hydrogenases. As the specific hydrogenase activities of the recombinant enzymes are lower (16–45%) than those of native hydrogenases, one can speculate that an ineffective maturation of the recombinant enzymes is responsible.

### 4.2. Recombinant Hydrogenase Production in the Presence of Specific Accessory Proteins

The composition of hydrogenase operons is mostly conserved and exhibits a high degree of similarity; however, the high specificity of the *cis*-acting maturation system to the corresponding hydrogenase is a barrier that makes heterologous production more challenging [139]. Lenz et al. constructed a broad-host-range plasmid carrying the entire membrane-bound hydrogenase (MBH) operon of *R. eutropha* H16 encompassing 21 genes. In fact, hydrogenase activity was observed when this plasmid was transferred to the hydrogenase-free host, *Pseudomonas stutzeri*, indicating the presence of fully assembled and functional MBH [128]. This successful heterologous MBH production implied that an entire operon including structural genes (*hoxKGZ*), seven *hyp* genes (*hypABFCDEX*), MBH-specific accessory genes (*hoxMLOQRTV*) and a two-component regulatory system (*hoxABCJ*) are essential for producing MBH activity in heterologous systems [128].

Similarly, the regulatory hydrogenase (RH) of *R. eutropha* was produced in the cytoplasm of *E. coli* by coproduction of eight *R. eutropha* proteins, including HoxBC forming the RH heterodimer and the maturation proteins HypABFCDE [140]. However, the truncated *R. eutropha* HypF protein turned out to be nonfunctional in *E. coli*, and the *E. coli* HypF took over cyanide ligand synthesis for recombinant *Reu*RH, indicating substantial differences in HypF-mediated CN ligand biosynthesis [128,141,142]. As mentioned above, *E. coli* has been used for the functional production of *Pfu*SHI (four structural and nine maturation genes *hypABFCDE*, *hycI*, *slyD, frxA*) [125]. A remarkable novel finding of this study was that only the four structural genes and one gene encoding ferredoxin oxidoreductase A *(frxA*) from *P. furiosus* are required for production of functional *Pfu*SHI in *E. coli*. Their expression was induced under anaerobic conditions similar to the native hydrogenase-related genes of *E. coli*, suggesting that the *E coli* maturases can mediate post-translational maturation of an archaeal [NiFe]-hydrogenase.

In a metagenomics approach, Maroti et al. successfully produced a novel [NiFe]-hydrogenase from the Sargasso Sea in *Thiocapsa roseopersicina* by co-expressing only two accessory genes, *hyaD* and *hupH*, from *A. macleodii* [116]. The structural proteins showed 99% identity to the *A. macleodii* hydrogenase subunits HyaA and HyaB. This result emphasizes that the level of similarity of the hydrogenases appears to be more important than the similarity of the maturation apparatuses for the successful production of an active hydrogenase in a foreign host. However, the activity of the recombinant environmental hydrogenase was only 15%–20% of that of the native *T. roseopersicina* hydrogenase. The low homology between accessory maturation proteins may be the reason for the low activity of the recombinantly produced hydrogenases without co-expression of specific maturases in the expression system.

We anticipate that synthetic biology approaches will allow for the high-level production of recombinant [NiFe]-hydrogenase more easily. In fact, Schiffels et al. [130] have used an innovative cloning platform to achieve high-yield production and maturation of the SH from *R. eutropha* H16 in *E. coli*. Using this cloning platform, each gene received its own T7 promoter and terminator. The genes were placed on two different vectors, one comprising the structural genes *hoxFUYHI* as well as the specific endopeptidase *hoxW* and the second harboring *hypC1D1E1A2B2F2X*, encoding the maturases. Further addition of *R. eutropha hoxN1*, encoding a high-affinity nickel permease, increased maturation efficiency in *E. coli.* Compared to previously reported SH production in the native host *R. eutropha*, the authors obtained a 3-fold increase in both protein yield and specific activity in whole cells. The recombinant SH was isolated from *E. coli* cells grown under both aerobic and anaerobic conditions with the same purity and stability. Remarkably, protein purified from anaerobically grown cells showed a 1.8-fold higher specific hydrogenase activity when compared to native SH isolated from the native host *R. eutropha* [130,143]. Thus, this platform based on synthetic biology provides a novel direction to produce recombinant [NiFe]-hydrogenases and facilitate directed evolution approaches to optimize the enzymes and their reaction conditions. However, current methods still have low throughput for measuring hydrogenase activity, which is a major limit for research studies and biotechnological applications. Lacasse et al. [144] developed a whole-cell colorimetric hydrogenase activity assay suitable for high-throughput applications based on the reduction of benzyl viologen with a very high specificity. This assay is a promising method for future screenings of growth conditions and factors facilitating the heterologous production of active [NiFe] hydrogenases.

### 4.3. In Vitro Reconstitution Systems for [NiFe]-Hydrogenases

The in vitro reconstitution of [FeFe]-hydrogenases by outfitting the apo-enzyme with a chemically synthesized metal cofactor enhanced yield and activity and also greatly facilitated the investigation of unknown [FeFe]-hydrogenases from various origins (see above). Development of a similar in vitro reconstitution strategy for [NiFe] hydrogenases is much more complicated because of the higher complexity of the active site architecture and the bipartite nature of the hydrogenase module. So far, different studies attempted to in vitro reconstitute catalytic active [NiFe]-hydrogenase by mixing only purified independently produced proteins (Figure 3B). Although the mechanism of small subunit maturation is not well understood, the FeS clusters are often stably incorporated into the apo-protein by the host’s Isc/Suf machineries during heterologous small subunit production in *E. coli* [11,24,112,139]. The catalytic activity of the NAD^+^-reducing SH (HoxFUYH) from *R. eutropha* could be reconstituted by mixing cell extracts containing the sub-modules of the enzyme. This strategy led to the recovery of 72% of the enzymatic activity of the native SH [131], indicating that the HoxHY and HoxFU are synthesized as stable and functional sub-modules.

A little earlier, an in vitro maturation system for the large subunit HycE of *E. coli* hydrogenase 3 (Hyd-3) was described that was based on mixing extracts of nickel-free HycE precursor, HypBCDEF and HycI in the presence of nickel to generate an active enzyme (12% of the wild-type) under anaerobic conditions [123]. Recently, Soboh and coworkers mixed extracts containing the precursors of Hyd-2 from *E. coli* in vitro with the purified HybG-HybDE that serves as assembly site and carrier of the [Fe(CN)_2_(CO)] unit of the catalytic center [122,145]. They obtained catalytically active [NiFe]-hydrogenases, which gained further activity upon addition of purified HypF and HypE [122]. Furthermore, the addition of an “activation mixture” including reductant, ATP, nickel and carbamoyl phosphate was necessary, suggesting that at least a part of the reconstitution process was mimicking the catalytic in vivo biosynthesis process.

Taken together, these findings strongly suggest that it is possible to isolate incompletely processed intermediates during the maturation process and to utilize these as a basis for the in vitro maturation of apo-[NiFe]-hydrogenase. The studies described above advanced our understanding of how these accessory proteins work together in active site assembly. Moreover, this innovative in vitro maturation system for [NiFe]-hydrogenases provides an appealing strategy to engineer hydrogenases with desired properties, e.g., improved O_2_ tolerance or incorporation of alternative catalytic metal ion centers, which, in turn, offers the possibility for creating new enzymatic reactions.

At present, the investigation of the isolated large subunits of [NiFe]-hydrogenases stands in the focus of research to understand the role of the individual subunits in the H_2_ activation process. In fact, it was demonstrated that the isolated large subunit of the membrane-bound hydrogenase of *R. eutropha* was capable in H_2_ activation as it showed catalytic hydrogen/deuterium exchange activity even in the presence of O_2_ [127,146,147,148]. Such a size reduction, that is the removal of the FeS cluster containing a small subunit, greatly facilitates spectroscopic investigation of the catalytic center, as demonstrated for the recombinantly overproduced large subunit of the regulatory [NiFe]-hydrogenase of *R. eutropha* [149]. Notably, the cofactor-free apo-large subunits protein provide a suitable target for semiartificial reconstitution trials with chemically synthesized Fe-cofactor compounds in conjunction with separate insertion of nickel irons.

Despite of the fact that considerable progress has been made in the heterologous production of [NiFe]-hydrogenases, most recombinant enzymes have significantly lower specific activities than their native counterparts, indicating that the hydrogenase maturation efficiency within the heterologous host cells is still limiting. Therefore, the complex process of post-translational maturation of [NiFe]-hydrogenases requires further exploration.

## 5. Biohydrogen Production through Heterologous Gene Expression

Hydrogen is considered a promising alternative to classical fossil fuels owing to its various merits. Despite the fact that current H_2_ production mainly depends on fossil fuels [150,151,152,153], biological approaches based on photosynthetic and fermentative processes have been greatly developed to generate biohydrogen in a sustainable way. These biological approaches mainly aim at the heterologous production of hydrogenases but also the production of other hydrogen-generating enzymes, such as nitrogenases [154,155,156], modified photosystem I [157,158,159], or semisynthetic catalysts composed of a chemically synthesized metal catalysts and a recombinantly produced protein [160,161,162]. Here, the fermentative hydrogen production is generally more efficient than the photosynthetic one because of its numerous benefits: (i) independence from the availability of light in dark fermentation [163,164]; (ii) higher H_2_ production rates [165,166]; (iii) use of a wide range of carbon sources (more attractively from wastes); (iv) requirement of less energy; and (v) technical much simpler and more stable process [167,168,169].

In biological H_2_ production processes, photosynthetic microorganisms collect the energy of sunlight and use it to activate special hydrogen-producing enzymes such as hydrogenases or nitrogenases in biophotolysis or photofermentation. So far, a number of strategies have been afforded to use these organisms in H_2_ production [170,171]. Sulfur or magnesium deprivation results in the inactivation of photosystem II and subsequently reduced O_2_ evolution in order to protect the O_2_-sensitive cyanobacterial [FeFe] hydrogenases and improves hydrogen production [172,173] More recently, co-production of cyanoglobin GlbN from *Nostoc commune* was used to protect the heterologously produced *C. acetobutylicum* [FeFe]-hydrogenase HydA from oxidation when produced in *Nostoc PCC7120*. This resulted in an increased H_2_ yield of about 20-fold under aerobic conditions [174]. Additionally, the reduction of antennas was successfully used to enlarge the utilized spectrum and the quantity of the captured light at high-light intensities, thereby improving the light efficiency [175,176]. Similarly, metabolic and genetic engineering were applied for improvement of the H_2_ yield in dark fermentation [177,178,179]. Nevertheless, the sensitivity of hydrogenases to O_2_, their poor catalytic efficiency for H_2_ production as well as the low H_2_ yield on substrates in dark fermentation are the major obstacles to develop H_2_ production systems using photosynthetic microorganisms [180,181,182,183].

Up to date, several improvements in biohydrogen production have been made to overcome the current major obstacles of slow H_2_ production and low H_2_ yield in dark fermentation by heterologous hydrogenase production with the aid of the native *E. coli* maturation machinery. At the earliest, H_2_ production was enhanced by 3-times through recombinant production of *C. butyricum* [FeFe]-hydrogenase in an *E. coli* mutant lacking a native hydrogenase activity [184]. A similar improvement was accomplished by overproducing recombinant HydA from *Ethanoligenes harbinenese* in non-hydrogen producing *E. coli* BL21 [86]. Furthermore, recombinant *Rhodobacter sphaeroides* HupSL hydrogenase in *E. coli* produced 200-fold more H_2_ than wild-type *R. sphaeroides* cells under dark anaerobic conditions [185]. Akhtar and collegues constructed a synthetic YdbK-dependent pyruvate:H_2_ pathway in *E. coli* BL21(DE3) by co-producing six proteins, including *E. coli* YdbK, *C. pasteurianum* [4Fe4S]-ferredoxin and *C. acetobutylicum* HydEFGA [186,187]. The deletion of *iscR* and/or the addition of thiamine pyrophosphate to the medium enhanced both total YdbK activity and H_2_ yield per glucose (19 µmol H_2_/(h·mg)). Additional co-production of *B. subtilis* α-amylase, AmyE, enabled starch-dependent H_2_ production in *E. coli* BL21 [65,187]. In another approach, the [FeFe]-hydrogenase HydA from *Enterobacter cloacae* was employed in *E. coli* BL21. Here, recombinant HydA production increased the H_2_ yield 1.4-fold compared to the wild type *E. cloacae* strain [188,189]. Maeda et al. produced the cyanobacterial [NiFe]-hydrogenase HoxEFUYH from *Synechocystis* sp. PCC 6803 together with its maturation factors HypABFCDE and HoxW in *E. coli*, resulting in a 41-fold higher H_2_ production (10 µmol H_2_/(h·mg)) compared to *E. coli* producing only its native hydrogenase 3 [190]. Interestingly, single deletion of any of the seven cyanobacterial hydrogenase maturation factors HypABFCDE and HoxW affected both hydrogenase activity and H_2_ production in a hydrogenase negative *E. coli* strain to varying degrees [133]. All seven factors show a high degree of specificity towards their particular hydrogenase target and are required for optimal hydrogenase maturation. Nevertheless, only two of them, HypA and HoxW, need to be co-expressed, since their function in activating the cyanobacterial hydrogenase cannot be taken over by their *E. coli* homologues [109].

In contrast to cyanobacteria, *E. coli* cannot utilize light energy directly. Nevertheless, several attempts have been made to make light energy available for *E. coli* to be used for the production of hydrogen. On the one hand, it has been shown that protons generated by rhodopsin can migrate along the cytoplasmic membrane [191]. These protons could serve as a substrate for H_2_ production. In fact, Kim et al. introduced genes for the synthesis of proteorhodopsin and retinal in addition to a [NiFe]-hydrogenase encoding gene from *H. marinus* into *E. coli* BL21(DE3) for light-driven biohydrogen production. The presence of proteorhodopsin and retinal increased the hydrogen production ~1.3-fold (4.25 µmol H_2_/(h·mg)) compared to the hydrogenase only strain [192]. On the other hand, the use of bioinorganic hybrid systems, comprised of a semiconductor and hydrogenase-producing bacterial cells, can be used [193]. This strategy was successfully applied to engineer *E. coli* cells that synthesizes a metal ion complex-binding protein on their surface that collects the light energy in addition to a hydrogenase that uses the solar energy for hydrogen production [15]. Moreover, the additional encapsulation of hydrogen-producing bacteria within a biomimetic silica matrix allowed us to use O_2_-sensitive hydrogenases, even under aerobic conditions [15]. However, compared to the activity of purified hydrogenases, the hydrogen yield that can be obtained from these bioinorganic hybrid systems is very low (0.5 µmol H_2_/10^8^ cells within 36 h) [15], and it requires the use of hazardous heavy metal ion complexes.

Another example is the membrane-bound [NiFe]-hydrogenase, HupSL, from the photoautotrophic bacterium *Rhodopseudomonas palustris* that plays a key role in the oxidation of H_2_ produced as a side product in the nitrogenase reaction [194] in *R. palustris*. Zhou et al. [195] used this hydrogenase for the construction of an engineered *E. coli* BL21(DE3) strain with remarkably enhanced H_2_ production activity (2.23 µmol H_2_/(h·mg)).

However, the research on H_2_ production based on microbial biotechnology is currently still in its infancy. Both the maturity of the technology and the production scale are far away from meeting the requirements of commercial production. Therefore, significant improvements in H_2_ production rates as well as yields in engineered *E. coli* strains are required.

## 6. Conclusions

Over the past few years, enormous progress has been made in our understanding of hydrogenase structure, function and their applications in biohydrogen production in vivo or in vitro mediated by recombinantly produced enzymes. However, despite these progresses, still the state of the production of recombinant hydrogenases is not satisfactory with regard to potential applications. Most recombinant hydrogenases have not yet reached the catalytic properties of natural enzymes and exhibit O_2_-sensitivity of their active site which needs to be protected. For the potential industrial applications of these enzymes, more efficient production systems need to be developed that produce recombinant hydrogenases with a larger quantity, higher specific activity and improved O_2_ tolerance. Nevertheless, work on [NiFe]-hydrogenases in particular has shown that many microbes have developed a natural solution to confer these enzymes’ O_2_ tolerance and catalytic bias. For example, a variety of production systems for *R. eutropha* H16 [NiFe]-hydrogenases have been succeeded in different organisms [196]. However, our ability to produce these O_2_-tolerant hydrogenases in heterologous hosts is still challenging, and this limitation extremely hinders the viable potentials in hydrogen production and fuel cells. An interesting attempt to circumvent the O_2_ sensitivity is to use specifically designed low-potential viologen-modified redox polymers, thus providing a protection matrix against oxidative damage and high-potential deactivation of O_2_-sensitive hydrogenases. Recent developments in the viologen-based polymer matrix offered the possibility of using O_2_-sensitive enzymes for H_2_ oxidation in an oxidative environment [197,198,199,200]. Hence, highly O_2_-sensitive catalysts can now be considered for applications under very harsh oxidative conditions in H_2_/O_2_ mixed feeding biofuel cells and other energy-converting devices.

In addition, the development of artificial in vitro maturation systems for hydrogenases in a great variety of different scaffolds could help to improve our fundamental understanding of hydrogenases, including the potential limiting factors, spectroscopic studies of the active center and the effects of O_2_ on the enzyme activity. The successful in vitro maturation system can provide catalytically active hydrogenase subunits used for protein–protein docking studies with enzymes demanding low-potential electrons such as formate dehydrogenase or CO dehydrogenase [127,201]. Indeed, this strategy facilitates their potential application in industrially useful H_2_-conversion catalysts. Moreover, another important direction of recombinant research is to promote the production of minimized artificial hydrogenase proteins, thus providing the original enzyme model for the development of hydrogenase chemical mimicry catalysts.

In order to facilitate their biotechnological application, the recombinant hydrogenase should ideally not only have high oxygen tolerance and long-term catalytic stability, but also possess enzymatic characteristics that are absent in natural hydrogenases, such as the ability to use cheap electron donors. Moreover, the biotechnological production process of these enzymes should be flexible with regard to the heterologous expression strain and the cultivation conditions without affecting product quality and quantity, which was not yet achieved due to the complicated maturation mechanisms.

We have discussed the efforts contributed to the in vivo and in vitro maturation in this short perspective. Undoubtedly, we hope the combined efforts of molecular biology, bioinformatics and synthetic biology may practically help to improve our understanding of the different catalytic mechanisms of hydrogenases and the molecular details of oxygen tolerance and maturation, as well as to find solutions to the imminent energy exhaustion by the development of a hydrogen economy in the future.

## Figures and Tables

**Table 1:** Activities of recombinantly produced [Fe]-hydrogenases.

Hydrogenase	Expression Host Cofactor	Hyd Yield [mg/g]	Specific Activity [U mg^−1^]	Ref.
*Dth*HmdII	*E. coli* + *Mma*Hmd FeGP	NR	8 ^a^	[35]
*Mja*Hmd	*M. jannaschii*	NR	350 ^§^	[31]
*Mja*HmdII	*E. coli* + *Mma*Hmd FeGP	NR	5 ^a^	[35]
*Mja*Hmd	*E. coli* + *Dth*HmdII FeGP	NR	42	[35]
*Mja*Hmd	*E. coli* + *Mma*Hmd FeGP	2.5	370–1100 ^§^	[31,36,37]
*Mka*Hmd	*M. kandleri*	0.4	360 ^§^	[32]
*Mka*Hmd	*E. coli* + *Mja*Hmd FeGP	NR	1100 *^,§^	[31]
[Fe]-*Mja*Hmd	*E. coli* + Fe(II)-complex	NR	2.5	[37]
[Mn]-*Mja*Hmd	*E. coli* + Mn(I)-complex	NR	1.5	[37]

For comparison, homologous productions of hydrogenases are highlighted in gray. NR: not reported; ^a^ = mU mg^−1^ -protein; * similar to the reconstituted *Mja*Hmd; ^§^ activity measurement at 65 °C. One unit (U) corresponds to the formation of 1 µmol of methenyl-H_4_MPT^+^ from methylene-H_4_MPT per min. *Dth: Desulfurobacterium thermolithotrophum; Mja: Methanocaldococcus jannaschii; Mka: Methanopyrus kandleri; Mma: Methanothermobacter marburgensis*.

**Table 2:** Activities of recombinantly produced [FeFe]-hydrogenases.

Hydrogenase	Host for Recombinant Production	Origin of the Maturation Proteins	Hyd Yield [mg/L]	Whole-Cell Activity	Specific Activity of Purified Enzyme	Ref.
*Cac*HydA	*C. acetobutylicum*	Host	NR	NR	162 ^b^	[63]
*Cac*HydA	*E. coli*	*C. acetobutylicum*	>1	96 ^a^	75 ^b^	[64]
*Cac*HydA	*E. coli ΔiscR*	*C. acetobutylicum*	0.003	1.3 ^a,^*	96 ^b^	[65]
*Cac*HydA	*S. elongatus*	*C. reinhardtii*	NR	NR	0.05 ^b^	[66]
*Cac*HydB	*E. coli*	*C. acetobutylicum*	NR	NR	8.6 ^b^	[64]
*Cbu*HydA	*E. coli*	Host	NR	500 ^a^	NR	[67]
*Cpa*HydA	*C. pasteurianum*	Host	NR	1681 ^a^	1236 ^b^	[68]
*Cpa*HydA	*in vitro*	*S. oneidensis*	NR	NA	242 ^b^	[69]
*Cpa*HydI	*E. coli*	*S. oneidensis*	8	NR	1087 ^b^	[70]
*Cpa*HydI	*in vitro*	*S. oneidensis*	NR	NA	1000 ^a^	[71]
*Cpa*HydI	*in vitro*	*S. oneidensis*	NR	NR	2000 ^b^	[72]
*Cpa*HydI	*in vitro*	-	NR	NR	2000 ^b^	[73]
*Cpa*HydI	*Synechococcus* sp.	Host	NR	NR	4.6 ^b^	[74]
*Cre*HydA1	*C. reinhardtii*	Host	0.07	13.8 ^b,^*	741 ^b^	[75]
*Cre*HydA1	*C. acetobutylicum*	Host	0.1–1.0	NR	625–760 ^b^	[76], [77]
*Cre*HydA1	*E. coli*	*C. reinhardtii*	low	NR	0.4 ^b^	[78]
*Cre*HydA1	*E. coli*	*C. acetobutylicum*	0.8–1.0	61^a^	150 ^b^	[64]
*Cre*HydA1-Fd	*E. coli*	*C. acetobutylicum*	NR	NR	1000 ^b^	[79]
*Cre*HydA1	*E. coli*	*S. oneidensis*	30	NR	641 ^b^	[70]
*Cre*HydA1	*in vitro*	*C. reinhardtii*	NR	NR	600 ^b^	[80]
*Cre*HydA1	*in vitro*	-	NR	NR	700 ^b^	[81]
*Cre*HydA1	*S. oneidensis*	Host	0.4–0.5	NA	740 ^b^	[82]
*Cre*HydA1	*Synechocystis* sp.	Host	NR	NR	0.1 ^b^	[83]
*Cre*HydA2	*E. coli*	*C. acetobutylicum*	0.8–1.0	108 ^a^	116 ^b^	[64]
*Csa*HydA	*in vitro*	*C. acetobutylicum*	NR	NR	2.5 ^b^	[84]
*Csu*HydA	*E. coli*	*S. oneidensis*	NR	NA	6.5 ^b^	[85]
*Eha*Hyd	*E. coli*	Host	NR	NR	70 ^b^	[86]
*Ehi*Hyd	*E. coli*	Host	NR	NR	0.04 ^b^	[87]
*Pgr*Hyd	*E. coli*	Host	NR	NR	2131 ^b^	[88]
*Sob*HydA1	*C. acetobutylicum*	Host	NR	NR	633 ^b^	[76]
*Son*HydA	*Anabaena* sp.	*S. oneidensis*	NR	NR	0.06 ^b^	[89]

For comparison, homologous productions of hydrogenases are highlighted in gray. NA: not applicable; NR: not reported; ^a^ = nmol H_2_ min^−1^ mL^−1^ culture; ^b^ = umol H_2_ min^−1^ mg^−1^-protein. * indicates activity measured from crude cell extracts. One unit of Hyd yield corresponds to 1 mg of the purified Hyd protein per 1 L culture. *Cac*: *Clostridium acetobutylicum*; *Cbu*: *Clostridium butylicum*, *Cpa*: *Clostridium pasteurianum*; *Cre*: *Chlamydomonas reinhardtii*, *Csa*: *Clostridium saccharobutylicum*; *Csu*: *Caldanaerobacter subterranus*; *Eha*: *Ethanoligenes harbinenese*; *Ehi*: *Entamoeba histolytica*; *Pgr*: *Pseudotrichonympha grassii*; *Sob*: *Scenedesmus obliquus*; *Son*: *Shewanella oneidensis*.

**Table 3:** Activities of recombinantly produced [NiFe]-hydrogenases.

Hydrogenase	Host for Recombinant Production	Origin of the Maturation Proteins	Hyd Yield	Whole-Cell Activity	Specific Activity of Purified Enzyme	Ref.
*Ama*HynSL	*A. macleodi ΔHynSL*	Host	NR	0.03 ^a,^*	0.1 ^c^	[113]
*Ama*HynSL	*E. coli*	*A. macleodii*	NR	3–70 × 10^−^^3 a,^*	NR	[114,115]
*Ama*HyaAB	*T. roseopersicina*	Host, *A. macleodii*	NR	5 × 10^−3 a^	NR	[116]
*Afl*HydSL	*E. coli*	Host	2.3 ^f^	NA	77 ^a^	[117]
*Dgi*HynAB	*D. gigas ΔHynAB*	Host	NR	1.9 ^a,^*	91 ^a^	[118]
*Dgi*HynAB	*D. fructosovorans ΔHynAB*	Host	NR	0.2 ^b^	NR	[119]
*Eco*Hyd1	*E. coli ΔHyd1*	Host	NR	4–7 × 10^−2 a,^*	1–3 × 10^−2 a^	[120,121]
*Eco* Hyd1/2	*in vitro*	*E. coli*	NR	NR	192 ^e^	[122]
*Eco*Hyd3 HycE	*in vitro*	*E. coli*	NR	NR	1.2 ^a^	[123]
*Hma*MBH	*E. coli*	Host	NR	0.07 ^a,^*	0.03 ^a^	[121]
*Npu*HupSL	*E. coli*	*N. punctiforme*	NR	208 ^a^	NR	[124]
*Pfu*SH	*E. coli*	*P. furiosus*	0.8 ^f^	2.9 ^a^	100 ^a^	[125]
*Reu*MBH	*R. eutropha* H16	Host	NR	1.0 ^c,^*	170 ^c^	[126]
*Reu*MBH preHoxG	*in vitro*	*E. coli*	NR	NR	2 × 10^−3 d^	[127]
*Reu*MBH	*in vitro*	*R. eutropha*	NR	NR	0.01 ^d^	[127]
*Reu*MBH	*P. stutzeri*	*R. eutropha*	NR	17–19 ^c,^*	NR	[128]
*Reu*RH	*E. coli*	*R. eutropha*	0.3 ^g^	NR	0.8 ^b^	[129]
*Reu*SH	*E. coli*	*R. eutropha*	0.4 ^g^	1.2 ^b,^*	230 ^b^	[130]
*Reu*SH	*in vitro*	*R. eutropha*	NR	NR	2.7 ^b^	[131]
*Rop*SH	*R. eutropha ΔSH ΔMBH*	Host, *R. opacus*	NR	5.9 ^a,^*	NR	[132]
*Syn*SH	*E. coli*	*Synechocystis* sp.	NR	0.04 ^a,^*	NR	[133]

For comparison, homologous productions of hydrogenases are highlighted in gray. NR: not reported; NA: no activity; ^a^ = µmol H_2_-evolved min^−1^ mg^−1^-protein by sodium dithionite-reduced methyl viologen; ^b^ = µmol H_2_-consumed min^−1^ mg^−1^-protein for reduction of NAD^+^; ^c^ = µmol H_2_-consumed min^−1^ mg^−1^-protein for reduction of methylene blue; ^d^ = s^−1^ D_2_ production rate; ^e^ = mU mL^−1^ reaction mixture; ^f^ = mg L^−1^-culture; g = mg g^−1^-wet mass; * indicates activity measured from crude cell extracts. *Ama*: *Alteromonas macleodii*; *Afl*: *Acetomicrobium flavidum*; *Dgi*: *Desulfovibrio gigas*; *Eco*: *Escherichia coli*; *Hma*: *Hydrogenovibrio marinus*; *Npu*: *Nostoc puncitiforme*; *Pfu*: *Pyrococcus furiosus*; *Reu*: *Ralstonia eutropha*; *Rop*: *Rhodococcus opacus*; *Syn*: Synechocystis sp. PCC6803.

## References

[1] Balat M. (2008). Potential importance of hydrogen as a future solution to environmental and transportation problems. Int. J. Hydrogen Energy.

[2] Dunn S. (2002). Hydrogen futures: Toward a sustainable energy system. Int. J. Hydrogen Energy.

[3] Shima S., Pilak O., Vogt S., Schick M., Stagni M.S., Meyer-Klaucke W., Warkentin E., Thauer R.K., Ermler U. (2008). The crystal structure sof [Fe]-hydrogenase reveals the geometry of the active site. Science.

[4] Crabtree G.W., Dresselhaus M.S. (2008). The hydrogen fuel alternative. MRS Bull..

[5] Zeng K., Zhang D. (2010). Recent progress in alkaline water electrolysis for hydrogen production and applications. Prog. Energy Combust. Sci..

[6] Ursua A., Gandia L.M., Sanchis P. (2012). Hydrogen production from water electrolysis: Current status and future trends. Proc. IEEE.

[7] Lubitz W., Ogata H., Rüdiger O., Reijerse E. (2014). Hydrogenases. Chem. Rev..

[8] Greening C., Biswas A., Carere C.R., Jackson C.J., Taylor M.C., Stott M.B., Cook G.M., Morales S.E. (2016). Genomic and metagenomic surveys of hydrogenase distribution indicate H_2_ is a widely utilised energy source for microbial growth and survival. ISME J..

[9] Vignais P.M., Billoud B. (2007). Occurrence, classification, and biological function of hydrogenases: An overview. Chem. Rev..

[10] Kalia V.C., Lal S., Ghai R., Mandal M., Chauhan A. (2003). Mining genomic databases to identify novel hydrogen producers. Trends Biotechnol..

[11] Forzi L., Sawers R.G. (2007). Maturation of [NiFe]-hydrogenases in *Escherichia coli*. BioMetals.

[12] Jugder B.-E., Welch J., Aguey-Zinsou K.-F., Marquis C.P. (2013). Fundamentals and electrochemical applications of [Ni–Fe]-uptake hydrogenases. RSC Adv..

[13] Edwards E.H., Bren K.L. (2020). Light-driven catalysis with engineered enzymes and biomimetic systems. Biotechnol. Appl. Biochem..

[14] Krassen H., Schwarze A., Friedrich B., Ataka K., Lenz O., Heberle J. (2009). Photosynthetic Hydrogen Production by a Hybrid Complex of Photosystem I and [NiFe]-Hydrogenase. ACS Nano.

[15] Wei W., Sun P., Li Z., Song K., Su W., Wang B., Liu Y., Zhao J. (2018). A surface-display biohybrid approach to light-driven hydrogen production in air. Sci. Adv..

[16] Chenevier P., Mugherli L., Darbe S., Darchy L., DiManno S., Tran P.D., Valentino F., Iannello M., Volbeda A., Cavazza C. (2013). Hydrogenase enzymes: Application in biofuel cells and inspiration for the design of noble-metal free catalysts for H_2_ oxidation. C. R. Chim..

[17] Mertens R., Greiner L., van den Ban E.C., Haaker H.B.C.M., Liese A. (2003). Practical applications of hydrogenase I from *Pyrococcus furiosus* for NADPH generation and regeneration. J. Mol. Catal. B Enzym..

[18] Ratzka J., Lauterbach L., Lenz O., Ansorge-Schumacher M.B. (2011). Systematic evaluation of the dihydrogen-oxidising and NAD + -reducing soluble [NiFe]-hydrogenase from *Ralstonia eutropha* H16 as a cofactor regeneration catalyst. Biocatal. Biotransform..

[19] Lauterbach L., Lenz O., Vincent K.A. (2013). H2-driven cofactor regeneration with NAD (P) +-reducing hydrogenases. FEBS J..

[20] Atomi H., Sato T., Kanai T. (2011). Application of hyperthermophiles and their enzymes. Curr. Opin. Biotechnol..

[21] Lojou E. (2011). Hydrogenases as catalysts for fuel cells: Strategies for efficient immobilization at electrode interfaces. Electrochim. Acta.

[22] Sargent F. (2016). The model [NiFe]-hydrogenases of *Escherichia coli*. Advances in Microbial Physiology.

[23] Mulder D.W., Shepard E.M., Meuser J.E., Joshi N., King P.W., Posewitz M.C., Broderick J.B., Peters J.W. (2011). Insights into [FeFe]-hydrogenase structure, mechanism, and maturation. Structure.

[24] Lacasse M.J., Zamble D.B. (2016). [NiFe]-hydrogenase maturation. Biochemistry.

[25] English C.M., Eckert C., Brown K., Seibert M., King P.W. (2009). Recombinant and in vitro expression systems for hydrogenases: New frontiers in basic and applied studies for biological and synthetic H_2_ production. Dalt. Trans..

[26] Huang G., Wagner T., Ermler U., Shima S. (2020). Methanogenesis involves direct hydride transfer from H_2_ to an organic substrate. Nat. Rev. Chem..

[27] Zirngibl C., van Dongen W., Schwörer B., von Bünau R., Richter M., Klein A., Thauer R.K. (1992). H2-forming methylenetetrahydromethanopterin dehydrogenase, a novel type of hydrogenase withaout iron-sulfur clusters in methanogenic archaea. Eur. J. Biochem..

[28] Hiromoto T., Ataka K., Pilak O., Vogt S., Stagni M.S., Meyer-Klaucke W., Warkentin E., Thauer R.K., Shima S., Ermler U. (2009). The crystal structure of C176A mutated [Fe]-hydrogenase suggests an acyl-iron ligation in the active site iron complex. FEBS Lett..

[29] Korbas M., Vogt S., Meyer-Klaucke W., Bill E., Lyon E.J., Thauer R.K., Shima S. (2006). The iron-sulfurcluster-free hydrogenase (Hmd) is a metalloenzyme with a novel iron binding motif. J. Biol. Chem..

[30] Schick M., Xie X., Ataka K., Kahnt J., Linne U., Shima S. (2012). Biosynthesis of the iron-guanylylpyridinol cofactor of [Fe]-hydrogenase in methanogenic archaea as elucidated by stable-isotope labeling. J. Am. Chem. Soc..

[31] Buurman G., Shima S., Thauer R.K. (2000). The metal-free hydrogenase from methanogenic archaea: Evidence for a bound cofactor. FEBS Lett..

[32] Ma K., Zirngibl C., Linder D., Stetter K.O., Thauer R.K. (1991). N5, N10-methylenetetrahydromethanopterin dehydrogenase (H2-forming) from the extreme thermophile *Methanopyrus kandleri*. Arch. Microbiol..

[33] Huang G., Wagner T., Ermler U., Bill E., Ataka K., Shima S. (2018). Dioxygen Sensitivity of [Fe]-Hydrogenase in the Presence of Reducing Substrates. Angew. Chem. Int. Ed..

[34] Lyon E.J., Shima S., Buurman G., Chowdhuri S., Batschauer A., Steinbach K., Thauer R.K. (2004). UV-A/blue-light inactivation of the “metal-free” hydrogenase (Hmd) from methanogenic archaea. Eur. J. Biochem..

[35] Watanabe T., Wagner T., Huang G., Kahnt J., Ataka K., Ermler U., Shima S. (2019). The bacterial [Fe]-hydrogenase paralog HmdII uses tetrahydrofolate derivatives as substrates. Angew. Chem. Int. Ed..

[36] Shima S., Schick M., Tamura H. (2011). Preparation of [Fe]-hydrogenase from methanogenic archaea. Methods in Enzymology.

[37] Pan H.J., Huang G., Wodrich M.D., Tirani F.F., Ataka K., Shima S., Hu X. (2019). A catalytically active [Mn]-hydrogenase incorporating a non-native metal cofactor. Nat. Chem..

[38] Thauer R.K., Kaster A.-K., Goenrich M., Schick M., Hiromoto T., Shima S. (2010). Hydrogenases from methanogenic archaea, nickel, a novel cofactor, and H_2_ Storage. Annu. Rev. Biochem..

[39] Lie T.J., Costa K.C., Pak D., Sakesan V., Leigh J.A. (2013). Phenotypic evidence that the function of the [Fe]-hydrogenase Hmd in Methanococcus maripaludis requires seven hcg (hmd co-occurring genes) but not hmdII. FEMS Microbiol. Lett..

[40] Fujishiro T., Tamura H., Schick M., Kahnt J., Xie X., Ermler U., Shima S. (2013). Identification of the HcgB Enzyme in [Fe]-Hydrogenase-Cofactor Biosynthesis. Angew. Chem..

[41] Fujishiro T., Bai L., Xu T., Xie X., Schick M., Kahnt J., Rother M., Hu X., Ermler U., Shima S. (2016). Identification of HcgC as a SAM-Dependent Pyridinol Methyltransferase in [Fe]-Hydrogenase Cofactor Biosynthesis. Angew. Chem..

[42] Bai L., Fujishiro T., Huang G., Koch J., Takabayashi A., Yokono M., Tanaka A., Xu T., Hu X., Ermler U. (2017). Towards artificial methanogenesis: Biosynthesis of the [Fe]-hydrogenase cofactor and characterization of the semi-synthetic hydrogenase. Faraday Discuss..

[43] Fujishiro T., Ermler U., Shima S. (2014). A possible iron delivery function of the dinuclear iron center of HcgD in [Fe]-hydrogenase cofactor biosynthesis. FEBS Lett..

[44] Fujishiro T., Kahnt J., Ermler U., Shima S. (2015). Protein-pyridinol thioester precursor for biosynthesis of the organometallic acyl-iron ligand in [Fe]-hydrogenase cofactor. Nat. Commun..

[45] Goldman A.D., Leigh J.A., Samudrala R. (2009). Comprehensive computational analysis of Hmd enzymes and paralogs in methanogenic Archaea. BMC Evol. Biol..

[46] Fujishiro T., Ataka K., Ermler U., Shima S. (2015). Towards a functional identification of catalytically inactive [Fe]-hydrogenase paralogs. FEBS J..

[47] Pan H.-J., Hu X. (2020). Biomimetic hydrogenation catalyzed by a manganese model of [Fe]-hydrogenase. Angew. Chem. Int. Ed..

[48] Yang X., Hall M.B. (2009). Monoiron Hydrogenase Catalysis: Hydrogen Activation with the Formation of a Dihydrogen, Fe−H^δ−^···H^δ+^−O, Bond and Methenyl-H_4_MPT^+^ Triggered Hydride Transfer. J. Am. Chem. Soc..

[49] Hedegård E.D., Kongsted J., Ryde U. (2015). Multiscale Modeling of the Active Site of [Fe] Hydrogenase: The H_2_ Binding Site in Open and Closed Protein Conformations. Angew. Chem. Int. Ed..

[50] Huang G., Wagner T., Wodrich M.D., Ataka K., Bill E., Ermler U., Hu X., Shima S. (2019). The atomic-resolution crystal structure of activated [Fe]-hydrogenase. Nat. Catal..

[51] Peters J.W., Schut G.J., Boyd E.S., Mulder D.W., Shepard E.M., Broderick J.B., King P.W., Adams M.W. (2015). [FeFe]- and [NiFe]-hydrogenase diversity, mechanism, and maturation. Biochim. Biophys. Acta—Mol. Cell Res..

[52] Horner D.S., Heil B., Happe T., Embley T.M. (2002). Iron hydrogenases—Ancient enzymes in modern eukaryotes. Trends Biochem. Sci..

[53] van der Giezen M., Tovar J., Clark C.G. (2005). Mitochondrion-derived organelles in protists and fungi. Int. Rev. Cytol..

[54] Melis A., Seibert M., Happe T. (2004). Genomics of green algal hydrogen research. Photosynth. Res..

[55] Meyer J. (2007). [FeFe] hydrogenases and their evolution: A genomic perspective. Cell. Mol. Life Sci..

[56] Peters J.W., Lanzilotta W.N., Lemon B.J., Seefeldt L.C. (1998). X-ray crystal structure of the Fe-only hydrogenase (CpI) from *Clostridium pasteurianum* to 1.8 angstrom resolution. Science.

[57] Vignais P. (2001). Classification and phylogeny of hydrogenases. FEMS Microbiol. Rev..

[58] Peters J.W. (1999). Structure and mechanism of iron-only hydrogenases. Curr. Opin. Struct. Biol..

[59] Nicolet Y., Cavazza C., Fontecilla-Camps J.C. (2002). Fe-only hydrogenases: Structure, function and evolution. J. Inorg. Biochem..

[60] Bortolus M., Costantini P., Doni D., Carbonera D. (2018). Overview of the Maturation Machinery of the H-Cluster of [FeFe]-Hydrogenases with a Focus on HydF. Int. J. Mol. Sci..

[61] Voordouw G., Hagen W.R., Krüse-Wolters M., van Berkel-Arts A., Veeger C. (1987). Purification and characterization of *Desulfovibrio vulgaris* (Hildenborough) hydrogenase expressed in *Escherichia coli*. Eur. J. Biochem..

[62] van Dongen W., Hagen Wilfred H., van den Berg W., Cees V. (1988). Evidence for an unusual mechanism of membrane translocation of the periplasmic hydrogenase of *Desulfovibrio vulgaris* (Hildenborough), as derived from expression in *Escherichia coli*. FEMS Microbiol. Lett..

[63] Demuez M., Cournac L., Guerrini O., Soucaille P., Girbal L. (2007). Complete activity profile of *Clostridium acetobutylicum* [FeFe]-hydrogenase and kinetic parameters for endogenous redox partners. FEMS Microbiol. Lett..

[64] King P.W., Posewitz M.C., Ghirardi M.L., Seibert M. (2006). Functional studies of [FeFe] hydrogenase maturation in an *Escherichia coli* biosynthetic system. J. Bacteriol..

[65] Akhtar M.K., Jones P.R. (2008). Deletion of *iscR* stimulates recombinant clostridial Fe-Fe hydrogenase activity and H_2_-accumulation in *Escherichia coli* BL21 (DE3). Appl. Microbiol. Biotechnol..

[66] Ducat D.C., Sachdeva G., Silver P.A. (2011). Rewiring hydrogenase-dependent redox circuits in cyanobacteria. Proc. Natl. Acad. Sci. USA.

[67] Subudhi S., Lal B. (2011). Fermentative hydrogen production in recombinant *Escherichia coli* harboring a [FeFe]-hydrogenase gene isolated from *Clostridium butyricum*. Int. J. Hydrogen Energy.

[68] Adams M.W.W., Mortenson L.E. (1984). The purification of hydrogenase II (uptake hydrogenase) from the anaerobic N_2_-fixing bacterium *Clostridium pasteurianum*. Biochim. Biophys. Acta—Bioenerg..

[69] Boyer M.E., Stapleton J.A., Kuchenreuther J.M., Wang C., Swartz J.R. (2008). Cell-free synthesis and maturation of [FeFe] hydrogenases. Biotechnol. Bioeng..

[70] Kuchenreuther J.M., Grady-Smith C.S., Bingham A.S., George S.J., Cramer S.P., Swartz J.R. (2010). High-yield expression of heterologous [FeFe] hydrogenases in *Escherichia coli*. PLoS ONE.

[71] Bingham A.S., Smith P.R., Swartz J.R. (2012). Evolution of an [FeFe] hydrogenase with decreased oxygen sensitivity. Int. J. Hydrogen Energy.

[72] Lampret O., Esselborn J., Haas R., Rutz A., Booth R.L., Kertess L., Wittkamp F., Megarity C.F., Armstrong F.A., Winkler M. (2019). The final steps of [FeFe]-hydrogenase maturation. Proc. Natl. Acad. Sci. USA.

[73] Esselborn J., Lambertz C., Adamska-Venkatesh A., Simmons T., Berggren G., Noth J., Siebel J., Hemschemeier A., Artero V., Reijerse E. (2013). Spontaneous activation of [FeFe]-hydrogenases by an inorganic [2Fe] active site mimic. Nat. Chem. Biol..

[74] Asada Y., Koike Y., Schnackenberg J., Miyake M., Uemura I., Miyake J. (2000). Heterologous expression of clostridial hydrogenase in the cyanobacterium *Synechococcus PCC7942*. Biochim. Biophys. Acta—Gene Struct. Expr..

[75] Kamp C., Silakov A., Winkler M., Reijerse E.J., Lubitz W., Happe T. (2008). Isolation and first EPR characterization of the [FeFe]-hydrogenases from green algae. Biochim. Biophys. Acta—Bioenerg..

[76] Girbal L., von Abendroth G., Winkler M., Benton P.M., Meynial-Salles I., Croux C., Peters J.W., Happe T., Soucaille P. (2005). Homologous and heterologous overexpression in *Clostridium acetobutylicum* and characterization of purified clostridial and algal Fe-only hydrogenases with high specific activities. Appl. Environ. Microbiol..

[77] von Abendroth G., Stripp S., Silakov A., Croux C., Soucaille P., Girbal L., Happe T. (2008). Optimized over-expression of [FeFe] hydrogenases with high specific activity in *Clostridium acetobutylicum*. Int. J. Hydrogen Energy.

[78] Posewitz M.C., King P.W., Smolinski S.L., Zhang L., Seibert M., Ghirardi M.L. (2004). Discovery of two novel radical S-adenosylmethionine proteins required for the assembly of an active [Fe] hydrogenase. J. Biol. Chem..

[79] Yacoby I., Tegler L.T., Pochekailov S., Zhang S., King P.W. (2012). Optimized Expression and Purification for High-Activity Preparations of Algal [FeFe]-Hydrogenase. PLoS ONE.

[80] Stapleton J.A., Swartz J.R. (2010). A cell-free microtiter plate screen for improved [FeFe] hydrogenases. PLoS ONE.

[81] Berggren G., Adamska A., Lambertz C., Simmons T.R., Esselborn J., Atta M., Gambarelli S., Mouesca J.-M., Reijerse E., Lubitz W. (2013). Biomimetic assembly and activation of [FeFe]-hydrogenases. Nature.

[82] Sybirna K., Antoine T., Lindberg P., Fourmond V., Rousset M., Mejean V., Bottin H. (2008). *Shewanella oneidensis*: A new and efficient System for Expression and Maturation of heterologous [Fe-Fe] Hydrogenase from *Chlamydomonas reinhardtii*. BMC Biotechnol..

[83] Berto P., D’apos Adamo S., Bergantino E., Vallese F., Giacometti G.M., Costantini P. (2011). The cyanobacterium S*ynechocystis sp. PCC 6803* is able to express an active [FeFe]-hydrogenase without additional maturation proteins. Biochem. Biophys. Res. Commun..

[84] McGlynn S.E., Ruebush S.S., Naumov A., Nagy L.E., Dubini A., King P.W., Broderick J.B., Posewitz M.C., Peters J.W. (2007). In vitro activation of [FeFe] hydrogenase: New insights into hydrogenase maturation. J. Biol. Inorg. Chem..

[85] Kelly C.L., Pinske C., Murphy B.J., Parkin A., Armstrong F., Palmer T., Sargent F. (2015). Integration of an [FeFe]-hydrogenase into the anaerobic metabolism of *Escherichia coli*. Biotechnol. Rep..

[86] Zhao X., Xing D., Zhang L., Ren N. (2010). Characterization and overexpression of a [FeFe]-hydrogenase gene of a novel hydrogen-producing bacterium *Ethanoligenens harbinense*. Int. J. Hydrogen Energy.

[87] Nixon J.E.J., Field J., McArthur A.G., Sogin M.L. (2003). Iron-dependent Hydrogenases of *Entamoeba histolytica* and *Giardia lamblia*: Activity of the Recombinant Entamoebic enzyme and evidence for lateral gene transfer. Biol. Bull..

[88] Inoue J.I., Saita K., Kudo T., Ui S., Ohkuma M. (2007). Hydrogen production by termite gut protists: Characterization of iron hydrogenases of parabasalian symbionts of the termite *Coptotermes formosanus*. Eukaryot. Cell.

[89] Gärtner K., Lechno-Yossef S., Cornish A.J., Wolk C.P., Hegg E.L. (2012). Expression of *Shewanella oneidensis* MR-1 [FeFe]-hydrogenase genes in *Anabaena* sp. strain PCC 7120. Appl. Environ. Microbiol..

[90] Atta M., Meyer J. (2000). Characterization of the gene encoding the [Fe]-hydrogenase from Megasphaera elsdenii. Biochim. Biophys. Acta—Protein Struct. Mol. Enzymol..

[91] Gorwa M.F., Croux C., Soucaille P. (1996). Molecular characterization and transcriptional analysis of the putative hydrogenase gene of *Clostridium acetobutylicum* ATCC 824. J. Bacteriol..

[92] Nagy L.E., Meuser J.E., Plummer S., Seibert M., Ghirardi M.L., King P.W., Ahmann D., Posewitz M.C. (2007). Application of gene-shuffling for the rapid generation of novel [FeFe]-hydrogenase libraries. Biotechnol. Lett..

[93] Heidelberg J.F., Paulsen I.T., Nelson K.E., Gaidos E.J., Nelson W.C., Read T.D., Eisen J.A., Seshadri R., Ward N., Methe B. (2002). Genome sequence of the dissimilatory metal ion–reducing bacterium *Shewanella oneidensis*. Nat. Biotechnol..

[94] Birrell J.A., Rüdiger O., Reijerse E.J., Lubitz W. (2017). Semisynthetic hydrogenases propel biological energy research into a new era. Joule.

[95] De Lacey A.L., Fernández V.M., Rousset M., Cammack R. (2007). Activation and Inactivation of Hydrogenase Function and the Catalytic Cycle: Spectroelectrochemical Studies. Chem. Rev..

[96] Goldet G., Brandmayr C., Stripp S.T., Happa T., Cavazza C., Fontecilla-Camps J.C., Armstrong F.A. (2009). Electrochemical kinetic investigations of the reactions of [FeFe]-hydrogenases with carbon monoxide and oxygen: Comparing the importance of gas tunnels and active-site electronic/redox effects. J. Am. Chem. Soc..

[97] Stripp S.T., Goldet G., Brandmayr C., Sanganas O., Vincent K.A., Haumann M., Armstong F.A., Happe T. (2009). How oxygen attacks [FeFe] hydrogenases from photosynthetic organisms. Proc. Natl. Acad. Sci. USA.

[98] Ghirardi M.L., Posewitz M.C., Maness P.-C., Dubini A., Yu J., Seibert M. (2007). Hydrogenases and Hydrogen Photoproduction in Oxygenic Photosynthetic Organisms. Annu. Rev. Plant Biol..

[99] Stripp S.T., Happe T. (2009). How algae produce hydrogen—News from the photosynthetic hydrogenase. Dalt. Trans..

[100] Torzillo G., Scoma A., Faraloni C., Giannelli L. (2015). Advances in the biotechnology of hydrogen production with the microalga *Chlamydomonas reinhardtii*. Crit. Rev. Biotechnol..

[101] Giannelli L., Scoma A., Torzillo G. (2009). Interplay between light intensity, chlorophyll concentration and culture mixing on the hydrogen production in sulfur-deprived *Chlamydomonas reinhardtii* cultures grown in laboratory photobioreactors. Biotechnol. Bioeng..

[102] Cohen J., Kim K., King P., Seibert M., Schulten K. (2005). Finding gas diffusion pathways inproteins: Application to O_2_ and H_2_ transport in CpI [FeFe]-hydrogenase and the role of packing defects. Structure.

[103] Cohen J., Kim K., Posewitz M., Ghirardi M.L., Schulten K., Seibert M., King P. (2005). Molecular dynamics and experimental investigation of H_2_ and O_2_ diffusion in [Fe]-hydrogenase. Biochem. Soc. Trans..

[104] Boynton J.E., Gillham N.W., Harris E.H., Hosler J.P., Johnson A.M., Jones A.R., Randolph-Anderson B.L., Robertson D., Klein T.M., Shark K.B. (1988). Chloroplast transformation in *Chlamydomonas* with high velocity microprojectiles. Science.

[105] Blowers A.D., Bogorad L., Shark K.B., Sanford J.C. (1989). Studies on *Chlamydomonas* chloroplast transformation: Foreign DNA can be stably maintained in the chromosome. Plant Cell.

[106] Kindle K.L. (1998). High-frequency nuclear transformation of *Chlamydomonas reinhardtii*. Methods Enzymol..

[107] Lumbreras V., Stevens D.R., Purton S. (1998). Efficient foreign gene expression in *Chlamydomonas reinhardtii* mediated by an endogenous intron. Plant J..

[108] Fontecilla-Camps J.C., Amara P., Cavazza C., Nicolet Y., Volbeda A. (2009). Structure-function relationships of anaerobic gas-processing metalloenzymes. Nature.

[109] Ogata H., Lubitz W., Higuchi Y. (2009). [NiFe] hydrogenases: Structural and spectroscopic studies of the reaction mechanism. Dalt. Trans..

[110] Happe R.P., Roseboom W., Plerlk A.J., Albracht S.P.J., Bagley K.A. (1997). Biological activition of hydrogen. Nature.

[111] Pierik A.J., Roseboom W., Happe R.P., Bagley K.A., Albracht S.P.J. (1999). Carbon monoxide and cyanide as intrinsic ligands to iron in the active site of [NiFe]-hydrogenases. NiFe(CN)_2_CO, biology’s way to activate H_2_. J. Biol. Chem..

[112] Böck A., King P.W., Blokesch M., Posewitz M.C. (2006). Maturation of Hydrogenases. Adv. Microb. Physiol..

[113] Vargas W.A., Weyman P.D., Tong Y., Smith H.O., Xu Q. (2011). [NiFe] Hydrogenase from *Alteromonas macleodii* with unusual stability in the presence of oxygen and high temperature. Appl. Environ. Microbiol..

[114] Weyman P.D., Vargas W.A., Chuang R.Y., Chang Y., Smith H.O., Xu Q. (2011). Heterologous expression of *Alteromonas macleodii* and *Thiocapsa roseopersicina* [NiFe] hydrogenases in *Escherichia coli*. Microbiology.

[115] Yonemoto I.T., Matteri C.W., Nguyen T.A., Smith H.O., Weyman P.D. (2013). Dual organism design cycle reveals small subunit substitutions that improve [NiFe] hydrogenase hydrogen evolution. J. Biol. Eng..

[116] Maroti G., Tong Y., Yooseph S., Baden-Tillson H., Smith H.O., Kovacs K.L., Frazier M., Venter J.C., Xu Q. (2009). Discovery of [NiFe] hydrogenase genes in metagenomic DNA: Cloning and heterologous expression in *Thiocapsa roseopersicina*. Appl. Environ. Microbiol..

[117] Mura G.M., Pedroni P., Pratesi C., Galli G., Serbolisca L., Grandit G. (1996). The [Ni-Fe] hydrogenase from the thermophilic bacterium *Acetornicrobiurn flavidurn*. Microbiology.

[118] Hatchikian E.C., Bruschi M., Le Gall J. (1978). Characterization of the periplasmic hydrogenase from *Desulfovibrio gigas*. Biochem. Biophys. Res. Commun..

[119] Rousset M., Magro V., Forget N., Guigliarelli B., Belaich J.P., Hatchikian E.C. (1998). Heterologous expression of the *Desulfovibrio gigas* [NiFe] hydrogenase in *Desulfovibrio fructosovorans* MR400. J. Bacteriol..

[120] Kim J.Y., Jo B., Cha H. (2010). Production of biohydrogen by recombinant expression of [NiFe]-hydrogenase 1 in *Escherichia coli*. Microb. Cell Fact..

[121] Kim J.Y.H., Jo B.H., Cha H.J. (2011). Production of biohydrogen by heterologous expression of oxygen-tolerant *Hydrogenovibrio marinus* [NiFe]-hydrogenase in *Escherichia coli*. J. Biotechnol..

[122] Soboh B., Lindenstrauss U., Granish C., Javed M., Herzberg M., Thomas C., Stripp S.T. (2014). [NiFe]-hydrogenase maturation *in vitro*: Analysis of the roles of the HybG and HypD accessory proteins. Biochem. J..

[123] Maier T., Böck A. (1996). Generation of active [NiFe] hydrogenase in vitro from a nickel-free precursor form. Biochemistry.

[124] Raleiras P., Khanna N., Miranda H., Mészáros L.S., Krassen H., Ho F., Battchikova N., Aro E.-M., Magnuson A., Lindblad P. (2016). Turning around the electron flow in an uptake hydrogenase. EPR spectroscopy and in vivo activity of a designed mutant in HupSL from *Nostoc punctiforme*. Energy Environ. Sci..

[125] Sun J., Hopkins R.C., Jenney F.E., McTernan P.M., Adams M.W.W. (2010). Heterologous Expression and Maturation of an NADP-Dependent [NiFe]-Hydrogenase: A Key Enzyme in Biofuel Production. PLoS ONE.

[126] Schink B., Schlegel H.G. (1979). The membrane-bound hydrogenase of *Alcaligenes eutrophus*. I. Solubilization, purification, and biochemical properties. BBA—Enzymol..

[127] Hartmann S., Frielingsdorf S., Ciaccafava A., Lorent C., Fritsch J., Seibert E., Priebe J., Haumann M., Zebger I., Lenz O. (2018). O_2_ -tolerant H_2_ activation by an isolated large subunit of a [NiFe] hydrogenase. Biochemistry.

[128] Lenz O., Gleiche A., Strack A., Friedrich B. (2005). Requirements for heterologous production of a complex metalloenzyme: The membrane-bound [NiFe] hydrogenase. J. Bacteriol..

[129] Buhrke T., Lenz O., Krauss N., Friedrich B. (2005). Oxygen tolerance of the H_2_-sensing [NiFe] hydrogenase from *Ralstonia eutropha* H16 is based on limited access of oxygen to the active site. J. Biol. Chem..

[130] Schiffels J., Pinkenburg O., Schelden M., Aboulnaga E.H.A.A., Baumann M.E.M., Selmer T. (2013). A innovative cloning platform enables large-scale production and maturation of an oxygen-tolerant [NiFe]-hydrogenase from *Cupriavidus necator* in *Escherichia coli*. PLoS ONE.

[131] Massanz C., Schmidt S., Friedrich B. (1998). Subforms and in vitro reconstitution of the NAD-reducing hydrogenase of *Alcaligenes eutrophus*. J. Bacteriol..

[132] Porthun A., Bernhard M., Friedrich B. (2002). Expression of a functional NAD-reducing [NiFe] hydrogenase from the gram-positive *Rhodococcus opacus* in the gram-negative *Ralstonia eutropha*. Arch. Microbiol..

[133] Wells M.A., Mercer J., Mott R.A., Pereira-Medrano A.G., Burja A.M., Radianingtyas H., Wright P.C. (2011). Engineering a non-native hydrogen production pathway into *Escherichia coli* via a cyanobacterial [NiFe] hydrogenase. Metab. Eng..

[134] Grzeszik C., Lübbers M., Reh M., Schlegel H.G. (1997). Genes encoding the NAD-reducing hydrogenase of *Rhodococcus opacus* MR11. Microbiology.

[135] Kaneko T., Sato S., Kotani H., Tanaka A., Asamizu E., Nakamura Y., Miyajima N., Hirosawa M., Sugiura M., Sasamoto S. (1996). Sequence Analysis of the Genome of the Unicellular Cyanobacterium *Synechocystis* sp. Strain PCC6803. II. Sequence Determination of the Entire Genome and Assignment of Potential Protein-coding Regions. DNA Res..

[136] Rousset M., Montet Y., Guigliarelli B., Forget N., Asso M., Bertrand P., Fontecilla-Camps J.C., Hatchikian E.C. (1998). [3Fe-4S] to [4Fe-4S] cluster conversion in *Desulfovibrio fructosovorans* [NiFe] hydrogenase by site-directed mutagenesis. Proc. Natl. Acad. Sci. USA.

[137] Báscones E., Imperial J., Ruiz-Argüeso T., Palacios J.M. (2000). Generation of new hydrogen-recycling *Rhizobiaceae* strains by introduction of a novel hup minitransposon. Appl. Environ. Microbiol..

[138] Raleiras P., Kellers P., Lindblad P., Styring S., Magnuson A. (2013). Isolation and characterization of the small subunit of the uptake hydrogenase from the cyanobacterium *Nostoc punctiforme*. J. Biol. Chem..

[139] Casalot L., Rousset M. (2001). Maturation of the [NiFe] hydrogenases. Trends Microbiol..

[140] Kleihues L., Lenz O., Bernhard M., Buhrke T., Friedrich B. (2000). The H_2_ sensor of *Ralstonia eutropha* is a member of the subclass of regulatory [NiFe] hydrogenases. J. Bacteriol..

[141] Burgdorf T., Lenz O., Buhrke T., van der Linden E., Jones A.K., Albracht S.P., Friedrich B. (2005). [NiFe]-hydrogenases of *Ralstonia eutropha* H16: Modular enzymes for oxygen-tolerant biological hydrogen oxidation. J. Mol. Microbiol. Biotechnol..

[142] Lenz O., Zebger I., Hamann J., Hildebrandt P., Friedrich B. (2007). Carbamoylphosphate serves as the source of CN-, but not of the intrinsic CO in the active site of the regulatory [NiFe]-hydrogenase from *Ralstonia eutropha*. FEBS Lett..

[143] Van der Linden E., Burgdorf T., de Lacey A.L., Buhrke T., Scholte M., Fernandez V.M., Friedrich B., Albracht S.P. (2006). An improved purification procedure for the soluble [NiFe]-hydrogenase of *Ralstonia eutropha*: New insights into its (in) stability and spectroscopic properties. J. Biol. Inorg. Chem..

[144] Lacasse M.J., Sebastiampillai S., Côté J.-P., Hodkinson N., Brown E.D., Zamble D.B. (2019). A whole-cell, high-throughput hydrogenase assay to identify factors that modulate [NiFe]-hydrogenase activity. J. Biol. Chem..

[145] Soboh B., Krüger S., Kuhns M., Pinske C., Lehmann A., Sawers R.G. (2010). Development of a cell-free system reveals an oxygen-labile step in the maturation of [NiFe]-hydrogenase 2 of *Escherichia coli*. FEBS Lett..

[146] Vignais P.M. (2005). H/D exchange reactions and mechanistic aspects of the hydrogenases. Coord. Chem. Rev..

[147] Fontecilla-Camps J.C., Frey M., Garcin E., Hatchikian C., Montet Y., Piras C., Vernede X., Volbeda A. (1997). Hydrogenase: A hydrogen-metabolizing enzyme. What do the crystal structures tell us about its mode of action?. Biochimie.

[148] Albracht S.P.J. (1994). Nickel hydrogenases: In search of the active site. BBA—Bioenerg..

[149] Caserta G., Lorent C., Ciaccafava A., Keck M., Breglia R., Greco C., Limberg C., Hildebrandt P., Cramer S.P., Zebger I. (2020). The large subunit of the regulatory [NiFe]-hydrogenase from *Ralstonia eutropha*—A minimal hydrogenase?. Chem. Sci..

[150] Gallucci F., Comite A., Capannelli G., Basile A. (2006). Steam reforming of methane in a membrane reactor: An industrial case study. Ind. Eng. Chem. Res..

[151] de Jong W. (2008). Sustainable Hydrogen Production by Thermochemical Biomass Processing. Hydrogen Fuel.

[152] Chen W.-H., Syu Y.-J. (2010). Hydrogen production from water gas shift reaction in a high gravity (Higee) environment using a rotating packed bed. Int. J. Hydrogen Energy.

[153] Mohan S.V., Pandey A. (2013). Biohydrogen Production: an introduction. Biohydrogen.

[154] Barahona E., Jiménez-Vicente E., Rubio L.M. (2016). Hydrogen overproducing nitrogenases obtained by random mutagenesis and high-throughput screening. Sci. Rep..

[155] Krishnan A., Qian X., Ananyev G., Lun D.S., Dismukes G.C. (2018). Rewiring of Cyanobacterial Metabolism for Hydrogen Production: Synthetic Biology Approaches and Challenges. Synthetic Biology of Cyanobacteria.

[156] Milton R.D., Minteer S.D. (2019). Nitrogenase Bioelectrochemistry for Synthesis Applications. Acc. Chem. Res..

[157] Winkler M., Kawelke S., Happe T. (2011). Light driven hydrogen production in protein based semi-artificial systems. Bioresour. Technol..

[158] Utschig L.M., Soltau S.R., Tiede D.M. (2015). Light-driven hydrogen production from Photosystem I-catalyst hybrids. Curr. Opin. Chem. Biol..

[159] Martin B.A., Frymier P.D. (2017). A Review of Hydrogen Production by Photosynthetic Organisms Using Whole-Cell and Cell-Free Systems. Appl. Biochem. Biotechnol..

[160] Simmons T.R., Berggren G., Bacchi M., Fontecave M., Artero V. (2014). Mimicking hydrogenases: From biomimetics to artificial enzymes. Coord. Chem. Rev..

[161] Caserta G., Roy S., Atta M., Artero V., Fontecave M. (2015). Artificial hydrogenases: Biohybrid and supramolecular systems for catalytic hydrogen production or uptake. Curr. Opin. Chem. Biol..

[162] Bren K.L. (2015). Multidisciplinary approaches to solar hydrogen. Interface Focus.

[163] Benemann J. (1996). Hydrogen Production: Progress and prospects. Nat. Biotechnol..

[164] Hallenbeck P. (2002). Biological hydrogen production; fundamentals and limiting processes. Int. J. Hydrogen Energy.

[165] Vignais P.M. (2008). Hydrogenases and H^+^-reduction in primary energy conservation. Results and Problems in Cell Differentiation.

[166] Vardar-Schara G., Maeda T., Wood T.K. (2008). Metabolically engineered bacteria for producing hydrogen via fermentation. Microb. Biotechnol..

[167] Chen X., Sun Y., Xiu Z., Li X., Zhang D. (2006). Stoichiometric analysis of biological hydrogen production by fermentative bacteria. Int. J. Hydrogen Energy.

[168] Levin D., Islam R., Cicek N., Sparling R. (2006). Hydrogen production by *Clostridium thermocellum* 27405 from cellulosic biomass substrates. Int. J. Hydrogen Energy.

[169] Ust’ak S., Havrland B., Muñoz J.O.J., Fernández E.C., Lachman J. (2007). Experimental verification of various methods for biological hydrogen production. Int. J. Hydrogen Energy.

[170] Chandrasekhar K., Lee Y.-J., Lee D.-W. (2015). Biohydrogen Production: Strategies to Improve Process Efficiency through Microbial Routes. Int. J. Mol. Sci..

[171] Zhang Q., Wang Y., Zhang Z., Lee D.J., Zhou X., Jing Y., Ge X., Jiang D., Hu J., He C. (2017). Photo-fermentative hydrogen production from crop residue: A mini review. Bioresour. Technol..

[172] Melis A. (2007). Photosynthetic H_2_ metabolism in *Chlamydomonas reinhardtii* (unicellular green algae). Planta.

[173] Volgusheva A.A., Jokel M., Allahverdiyeva Y., Kukarshikh G.P., Lukashev E.P., Lambreva M.D., Krendeleva T.E., Antal T.K. (2017). Comparative analyses of H_2_ photoproduction in magnesium- and sulfur-starved *Chlamydomonas reinhardtii* cultures. Physiol. Plant..

[174] Avilan L., Roumezi B., Risoul V., Bernard C.S., Kpebe A., Belhadjhassine M., Rousset M., Brugna M., Latifi A. (2018). Phototrophic hydrogen production from a clostridial [FeFe] hydrogenase expressed in the heterocysts of the cyanobacterium *Nostoc PCC 7120*. Appl. Microbiol. Biotechnol..

[175] Polle J.E.W., Kanakagiri S., Jin E.S., Masuda T., Melis A. (2002). Truncated chlorophyll antenna size of the photosystems—A practical method to improve microalgal productivity and hydrogen production in mass culture. Int. J. Hydrogen Energy.

[176] Singh H., Das D. (2018). Biofuels from Microalgae: Biohydrogen. Green Energy and Technology.

[177] Kim Y.M., Cho H.-S., Jung G.Y., Park J.M. (2011). Engineering the pentose phosphate pathway to improve hydrogen yield in recombinant *Escherichia coli*. Biotechnol. Bioeng..

[178] Wong Y.M., Wu T.Y., Ling T.C., Show P.L., Lee S.Y., Chang J.S., Ibrahim S., Juan J.C. (2018). Evaluating new bio-hydrogen producers: *Clostridium perfringens* strain JJC, *Clostridium bifermentans* strain WYM and *Clostridium sp*. strain Ade.TY. J. Biosci. Bioeng..

[179] Mishra P., Krishnan S., Rana S., Singh L., Sakinah M., Ab Wahid Z. (2019). Outlook of fermentative hydrogen production techniques: An overview of dark, photo and integrated dark-photo fermentative approach to biomass. Energy Strategy Rev..

[180] Hemschemeier A., Melis A., Happe T. (2009). Analytical approaches to photobiological hydrogen production in unicellular green algae. Photosynth. Res..

[181] Gutekunst K., Chen X., Schreiber K., Kaspar U., Makam S., Appel J. (2014). The Bidirectional NiFe-hydrogenase in *Synechocystis* sp. PCC 6803 Is Reduced by Flavodoxin and Ferredoxin and Is Essential under Mixotrophic, Nitrate-limiting Conditions. J. Biol. Chem..

[182] Bundhoo M.A.Z., Mohee R. (2016). Inhibition of dark fermentative bio-hydrogen production: A review. Int. J. Hydrogen Energy.

[183] Khetkorn W., Rastogi R., Incharoensakdi A., Lindblad P., Madamwar D., Pandey A., Larroche C. (2017). Microalgal hydrogen production—A review. Bioresour. Technol..

[184] Karube I., Urano N., Yamada T., Hirochika H., Sakaguchi K. (1983). Cloning and expression of the hydrogenase gene from *Clostridium butyricum* in *Escherichia coli*. FEBS Lett..

[185] Lee S.Y., Lee H.J., Park J.M., Lee J.H., Park J.S., Shin H.S., Kim Y.H., Min J. (2010). Bacterial hydrogen production in recombinant *Escherichia coli* harboring a HupSL hydrogenase isolated from *Rhodobacter sphaeroides* under anaerobic dark culture. Int. J. Hydrogen Energy.

[186] Akhtar M.K., Jones P.R. (2008). Engineering of a synthetic *hydF–hydE–hydG–hydA* operon for biohydrogen production. Anal. Biochem..

[187] Akhtar M.K., Jones P.R. (2009). Construction of a synthetic YdbK-dependent pyruvate: H_2_ pathway in *Escherichia coli* BL21 (DE3). Metab. Eng..

[188] Mishra J., Khurana S., Kumar N., Ghosh A.K., Das D. (2004). Molecular cloning, characterization, and overexpression of a novel [Fe]-hydrogenase isolated from a high rate of hydrogen producing *Enterobacter cloacae* IIT-BT 08. Biochem. Biophys. Res. Commun..

[189] Chittibabu G., Nath K., Das D. (2006). Feasibility studies on the fermentative hydrogen production by recombinant *Escherichia coli* BL21. Process Biochem..

[190] Maeda T., Vardar G., Self W.T., Wood T.K. (2007). Inhibition of hydrogen uptake in *Escherichia coli* by expressing the hydrogenase from the cyanobacterium *Synechocystis* sp. PCC 6803. BMC Biotechnol..

[191] Heberle J., Riesle J., Thiedemann G., Oesterhelt D., Dencher N.A. (1994). Proton migration along the membrane surface and retarded surface to bulk transfer. Nature.

[192] Kim J.Y., Jo B.H., Jo Y., Cha H.J. (2012). Improved production of biohydrogen in light-powered *Escherichia coli* by co-expression of proteorhodopsin and heterologous hydrogenase. Microb. Cell Fact..

[193] Honda Y., Hagiwara H., Ida S., Ishihara T. (2016). Application to Photocatalytic H_2_ Production of a Whole-Cell Reaction by Recombinant *Escherichia coli* Cells Expressing [FeFe]-Hydrogenase and Maturases Genes. Angew. Chem..

[194] Masukawa H., Sakurai H., Hausinger R.P., Inoue K. (2014). Sustained photobiological hydrogen production in the presence of N_2_ by nitrogenase mutants of the heterocyst-forming cyanobacterium *Anabaena*. Int. J. Hydrogen Energy.

[195] Zhou P., Wang Y., Gao R., Tong J., Yang Z. (2015). Transferring [NiFe] hydrogenase gene from *Rhodopeseudomonas palustris* into *E. coli* BL21(DE3) for improving hydrogen production. Int. J. Hydrogen Energy.

[196] Lenz O., Lauterbach L., Frielingsdorf S., Friedrich B. (2015). Oxygen-tolerant hydrogenases and their biotechnological potential. Biohydrogen.

[197] Plumeré N., Ruediger O., Oughli A.A., Williams R., Vivekananthan J., Poeller S., Schuhmann W., Lubitz W. (2014). A redox hydrogel protects hydrogenase from high-potential deactivation and oxygen damage. Nat. Chem..

[198] Fourmond V., Stapf S., Li H., Buesen D., Birrell J.A., Ruediger O., Lubitz W., Schuhmann W., Plumere N., Leger C. (2015). Mechanism of protection of catalysts supported in redox hydrogel films. J. Am. Chem. Soc..

[199] Oughli A.A., Ruff A., Boralugodage N.P., Rodriguez-Macia P., Plumere N., Lubitz W., Shaw W.J., Schuhmann W., Ruediger O. (2018). Dual properties of a hydrogen oxidation Ni-catalyst entrapped within a polymer promote self-defense against oxygen. Nat. Commun..

[200] Li H., Buesen D., Dementin S., Léger C., Fourmond V., Plumeré N. (2019). Complete Protection of O_2_-Sensitive Catalysts in Thin Films. J. Am. Chem. Soc..

[201] Rumpel S., Siebel J.F., Diallo M., Farès C., Reijerse E.J., Lubitz W. (2015). Structural Insight into the Complex of Ferredoxin and [FeFe] Hydrogenase from *Chlamydomonas reinhardtii*. ChemBioChem.

